# Trop-2 expression is associated with poor survival in pleural mesothelioma

**DOI:** 10.3389/fonc.2026.1841585

**Published:** 2026-05-29

**Authors:** Alejandro Aviles-Salas, Norma Hernández-Pedro, Enrique Caballé-Perez, José Lucio-Lozada, César Castillo-Ruiz, José Gregorio Chanona-Vilchis, Alejandro Aranda-Gutierrez, Mario Orozco-Morales, Pedro Barrios-Bernal, Rafael Parra-Medina, Luis Cabrera-Miranda, Andrés F. Cardona, Oscar Arrieta

**Affiliations:** 1 Pathology Department, Instituto Nacional de Cancerología (INCan), Mexico City, Mexico; 2 Personalized Medicine Laboratory, Instituto Nacional de Cancerología (INCan), Mexico City, Mexico; 3 Thoracic Oncology Unit, Instituto Nacional de Cancerología (INCan), Mexico City, Mexico; 4 Tecnologico de Monterrey, Oncology Institute and Breast Cancer Center, HospitalZambrano Hellion TecSalud, Nuevo León, Mexico; 5 Departament of Pathology, Instituto Nacional de Cancerología Bogotá, Bogotá, Colombia; 6 Thoracic Oncology Unit and Direction of Research and Education: Luis Carlos Sarmiento Angulo Cancer Treatment and Research Center, CTIC, Bogotá, Colombia

**Keywords:** chemotherapy, pleural mesothelioma, prognostic biomarkers, survival, trophoblast cell-surface antigen 2

## Abstract

**Introduction:**

Pleural mesothelioma (PM) lacks reliable biomarkers to guide therapy. Trophoblast cell-surface antigen 2 (Trop-2) is targetable in epithelial cancers, but its prevalence and clinical relevance in PM remain unclear.

**Methods:**

This retrospective cohort study selected patients with PM at the Instituto Nacional de Cancerología of Mexico from 2010 to 2022. Two independent pathologists evaluated Trop-2 positivity in histological tumor samples, defined as specific membranous staining of any intensity in ≥1% of analyzed tumor cells, equivalent to an H-score ranging 1 to 300. Median progression-free survival (mPFS) and overall survival (mOS) stratified by Trop-2 status were estimated by Kaplan–Meier method, and clinicopathologic associations were adjusted by multivariable Cox models.

**Results:**

Among sixty cases, Trop-2 was positive in 12 cases, associated with lung (*p* = 0.016) and non-regional lymph nodes (*p* = 0.002) metastases, shorter mOS (7.9 vs 27.8 months; *p* = 0.021), and independently predicted of worse PFS (adjusted hazard ratio [aHR] 2.20; *p* = 0.048) and OS (aHR 3.79; *p* = 0.009). Poorer OS was also predicted by Sarcomatoid histology (aHR 6.19; *p* = 0.025) and PLECH score >3 (aHR 2.64; *p* = 0.025).

**Discussion:**

Despite this small sample size, Trop-2 was related to adverse clinical outcomes, which may represent a key vulnerability for antibody-drug conjugates in PM, and highlight the need for its prospective standardized evaluation.

**Conclusions:**

Trop-2 was expressed in 20% of PMs, exclusively in epithelioid histology, associated with advanced disease and poor survival outcomes. These results support the prospective evaluation of Trop-2-targeted therapies in PM.

## 1 Introduction

Pleural mesothelioma (PM) is a rare but highly aggressive thoracic malignancy that originates from the mesothelial lining of the pleura. It represents approximately 0.17–0.25% of all cancer cases worldwide but contributes disproportionately to cancer-related morbidity and mortality (1, 2). The disease is strongly linked to occupational and environmental exposure to asbestos fibers, which may induce chronic pleural inflammation and cellular transformation decades after the initial exposure (3). Despite regulatory measures to restrict asbestos use, exposure remains prevalent in many low- and middle-income countries, including those in Latin America, where data on PM incidence and outcomes are limited and likely underreported (2, 4, 5).

PM typically presents at an advanced stage due to its insidious onset and non-specific clinical manifestations, including dyspnea, chest pain, and pleural effusion. Consequently, the prognosis has historically been poor, with a median overall survival (mOS) ranging from 6 to 13 months, depending on disease stage at diagnosis and access to treatment (6). Prognosis is further influenced by histologic subtype, with non-epithelioid variants (sarcomatoid and biphasic) associated with worse outcomes (7, 8).

The incorporation of immune checkpoint inhibitors (ICIs), including nivolumab, ipilimumab, and pembrolizumab, into first-line treatment regimens has modestly improved outcomes in PM, extending mOS to 17–22 months (9, 10). Nevertheless, prognosis remains generally poor. Although programmed death-ligand 1 (PD-L1) expression and clinical risk scores, such as the CALGB model, may serve as predictive and/or prognostic tools, most patients with PM exhibit limited responses to treatment, including ICIs (4). Moreover, although tumor mutational burden, specific gene mutations (e.g., *KMT2C* and *PBRM1*), and high expression of RRM1 and ERCC1 have been associated with longer survival, they have not been extensively validated as predictive markers (11, 12). These findings underscore the urgent need for novel clinical and molecular biomarkers to refine prognostic stratification and optimize treatment selection for patients with PM.

Trophoblast cell-surface antigen 2 (Trop-2) may address this unmet need given its critical role in tumor biology. Trop-2 is a transmembrane calcium signal transducer that regulates gene transcription and cell-cycle progression (13–15). Physiologically, it is expressed in epithelial tissues during fetal lung development and in specific adult stem cell compartments, but its expression is typically low or absent in most normal adult tissues (16). Conversely, Trop-2 overexpression has been reported in lung, breast, ovarian, prostate, and hepatocellular carcinomas, where it correlates with greater tumor aggressiveness, increased metastatic potential, and poorer clinical outcomes (13, 17).

Consequently, antibody–drug conjugates (ADCs) directed against Trop-2, such as sacituzumab govitecan and datopotamab deruxtecan, have shown significant antitumor activity in some malignancies, such as non–small cell lung cancer (NSCLC) and breast cancer (18–20). Similarly, preclinical evidence supports the antineoplastic potential of ADCs in PM (15), however, their clinical evaluation has been limited by the insufficient characterization of Trop-2 in this malignancy. This characterization is essential not only to define the therapeutic landscape for ADCs, but also to understand the broader biological impact of Trop-2. In other solid tumors, Trop-2 has already emerged as a potent driver of aggressiveness and a predictor of poor response to standard chemotherapy and immunotherapy (21, 22).

Despite its potential dual role as both a druggable target and a prognostic indicator, the prevalence and clinical significance of Trop-2 in PM remain poorly defined. Therefore, this study aims to determine the prevalence of Trop-2 expression in a retrospective cohort of patients with PM and to evaluate its association with clinicopathologic characteristics and survival outcomes.

## 2 Materials and methods

### 2.1 Study design

A retrospective, observational cohort study was conducted in patients diagnosed with PM between 2010 and 2022 at the Thoracic Oncology Unit of the Instituto Nacional de Cancerología (INCan), Mexico City. Eligible patients had histologically confirmed PM, available formalin-fixed paraffin-embedded (FFPE) tumor tissue for Trop-2 immunohistochemistry (IHC), and complete clinical data. Clinical and demographic information, including age, sex, smoking history, exposure to wood smoke or asbestos, Eastern Cooperative Oncology Group performance status (ECOG PS), clinical stage, histologic subtype, metastatic sites, baseline platelet and lactate dehydrogenase levels, and treatment regimens, was collected from electronic medical records. The PLECH score, a validated prognostic tool for PM, was calculated for each case based on five baseline variables: platelet count (+2 points), high LDH (+1 point), ECOG >2 (+1 point), chest pain at diagnosis (+2 points), and non-epithelioid histology (+1 point), resulting in a total score ranging from 0 to 7 (23). Consistent with previous validation, a score >3 was utilized to identify patients at high clinical risk. Patients without available FFPE tumor tissue or with a synchronous diagnosis of another malignancy were excluded from the analysis.

### 2.2 Ethical aspects

The Institutional Ethics and Scientific Board Review Committee approved this study (024/009/DGI) (CE|/008/24). Tissue samples were provided by the institutional pathology service. All personal data from enrolled patients were kept confidential using an internal number to identify tumor samples, and thereby, informed consent for this study was not applicable.

### 2.3 Immunohistochemical detection of Trop-2 expression

Tissue sections (3 μm thick) from FFPE blocks were obtained, deparaffinized, and stained with hematoxylin and eosin (H&E) to confirm the histopathologic diagnosis. Trop-2 expression was assessed by IHC. Endogenous peroxidase activity was quenched with hydrogen peroxide, followed by antigen retrieval in citrate buffer (BSB 0023; BioSB, Inc.) using a heat-induced epitope retrieval system. Sections were washed with 1× Tris-buffered saline (TBS, 40×) and incubated with a 1:200 dilution of mouse monoclonal anti–Trop-2 antibody (ENZ-ABS380; Enzo Life Sciences) at 37 °C for 45 minutes. Detection was performed using the MACH 4 Universal HRP Polymer Kit (M1U539; BioCare Medical), and the signal was visualized with diaminobenzidine (DAB) for 3 minutes. Finally, sections were counterstained with hematoxylin and briefly treated with ammonium hydroxide.

All specimens were initially reviewed by board-certified thoracic pathologists to confirm the diagnosis of PM and to ensure tissue adequacy for IHC analysis. Diagnosis was established according to current international guidelines, utilizing a standard panel of positive (e.g., calretinin, WT-1) and negative (e.g., TTF-1, p40) markers to differentiate PM from metastatic lung cancer. For each case, a representative FFPE block was selected based on the presence of at least 50% viable tumor cells, with careful exclusion of areas exhibiting significant necrosis or processing artifacts.

Subsequently, Trop-2 expression was independently evaluated by two board-certified pathologists specializing in oncologic pathology, who assessed both staining intensity and the percentage of positive tumor cells. Membranous staining intensity was categorized as follows: no staining (0), weak (1+, light brown), moderate (2+), or strong (3+, dark brown linear). For scoring consistency, strong staining (3+) was clearly visible under a 4× objective lens, moderate staining (2+) required a 10× lens, and weak staining (1+) was discernible only under a 40× lens. The histochemical score (H-score) was calculated by multiplying staining intensity by the percentage of positive cells, yielding a final score ranging from 0 to 300.

For the purpose of this study, Trop-2 positivity was defined as specific membranous staining of any intensity (1+, 2+, or 3+) in ≥1% of the analyzed tumor cells, equivalently to an H-score ranging 1 to 300. Since there was no validated scoring algorithm for Trop-2 immunohistochemistry established in pleural mesothelioma, H-score was independently scored as a raw number and not according to a prespecified cutoff. This approach provides a comprehensive initial characterization of Trop-2 expression, according to previous evidence in other neoplasms (19, 24). Isotype-matched IgG antibodies were used as negative controls, and a human breast carcinoma sample with confirmed Trop-2 expression served as the positive control.

### 2.4 Statistical analyses

Continuous variables were reported as means with standard deviations (SD) or medians with interquartile ranges (IQR), depending on data distribution assessed by the Kolmogorov-Smirnov test. Based on this distribution, group comparisons for continuous variables were performed using either the Student’s t-test or the Mann-Whitney U test. Categorical variables were summarized as frequencies and proportions, and group comparisons were conducted using the χ² test or Fisher’s exact test, as appropriate.

Survival outcomes were estimated using the Kaplan-Meier method, and differences between groups were assessed with the log-rank test. The impact of variables on progression-free survival (PFS) and overall survival (OS) was evaluated using univariate and multivariate Cox proportional hazards regression models to determine unadjusted (uHR) or adjusted hazard ratios (aHR). A two-sided *p*-value of <0.05 was considered statistically significant. All statistical analyses were performed using R (version 4.3.3) (25), employing the survival package (26).

## 3 Results

### 3.1 Clinical and pathologic features

A total of 80 patients with PM were identified. Of these, 18 patients were excluded due to incomplete clinical data or lack of available tissue for IHC, and 2 were excluded because of concomitant primaries (stage IV ovarian carcinosarcoma, n=1; stage IV melanoma, n=1). The final cohort included 60 patients, who were evaluated for clinicopathologic characteristics and survival outcomes (**Supplementary Figure 1**).

The overall cohort was predominantly male (76.7%), with a mean age at diagnosis of 63.50 years (IQR 18.2), absence of exposure to wood smoke (71.7%) or asbestos (65.0%), stage III-IV disease (73.3%), epithelioid histology (85.0%), and PLECH score >3 (51.7%), with a median PLECH score of 3.00 (IQR 3.0) (**Table 1**). The most frequent symptoms at diagnosis were chest pain (43.3%) and weight loss (36.7%).

**Table 1:** Clinical characteristics according to Trop-2 immunohistochemistry.

Clinical characteristics, n (%)	Overall n (%)	Trop-2 negative n (%)	Trop-2 positive n (%)	p value
	60 (100)	48 (80)	12 (20)	
Sex, n (%)				
Female	14 (23.3)	9 (18.8)	5 (41.7)	
Male	46 (76.7)	39 (81.2)	7 (58.3)	0.195
Age, (median [IQR])	63.50 [18.2]	63.50 [19.25]	63.50 [16.5]	0.934
Smoking status, n (%)				
Non-smoker	28 (46.7)	21 (43.8)	7 (58.3)	
Current/former	32 (53.3)	27 (56.2)	5 (41.7)	0.560
Passive smoking, n (%)				
Negative	50 (83.3)	40 (83.3)	10 (83.3)	
Positive	10 (16.7)	8 (16.7)	2 (16.7)	1.00*
Wood smoke exposure, n (%)				
Negative	43 (71.7)	35 (72.9)	8 (66.7)	
Positive	17 (28.3)	13 (27.1)	4 (33.3)	0.726*
Asbestos exposure, n (%)				
Absent	39 (65.0)	31 (64.6)	8 (66.7)	
Present	21 (35.0)	17 (35.4)	4 (33.3)	1.00*
ECOG, n (%)				
0-1	54 (90.0)	45 (93.8)	9 (75.0)	
≥2	6 (10.0)	3 (6.2)	3 (25.0)	0.088*
Clinical stage, n (%)				
I-II	16 (26.7)	16 (33.3)	0 (0.0)	
III-IV	44 (73.3)	32 (66.7)	12 (100.0)	**0.049**
Histology, n (%)				
Epithelioid	51 (85.0)	39 (81.2)	12 (100.0)	
Biphasic	1 (1.7)	1 (2.1)	0 (0.0)	
Sarcomatoid	5 (8.3)	5 (10.4)	0 (0.0)	
Non-classified	3 (5.0)	3 (6.2)	0 (0.0)	0.449
Pleural effusion, n (%)				
Absent	36 (60.0)	28 (58.3)	8 (66.7)	
Present	24(40.0)	20 (41.7)	4 (33.3)	0.843*
Metastases sites, n (%)***				
Contralateral pleura	18 (30.0)	16 (33.3)	2 (16.7)	0.317*
Lung	16 (26.7)	9 (18.8)	7 (58.3)	**0.016**
NRLN	13 (21.7)	6 (12.5)	7 (58.3)	**0.002**
Brain	1 (1.7)	1 (2.1)	0 (0.0)	1.00*
Adrenal	2 (3.3)	1 (2.1)	1 (8.3)	0.363*
Bone	11 (18.3)	9 (18.8)	2 (16.7)	1.00*
Liver	2 (3.3)	1 (2.1)	1 (8.3)	0.363*
First-line treatment, n (%)				
Chemotherapy only	43 (71.7)	36 (75.0)	7 (58.3)	
Multimodal therapy**	7 (11.7)	5 (10.4)	2 (16.7)	
Surgery only	2 (3.3)	2 (4.2)	0 (0.0)	
Best supportive care	8 (13.3)	5 (10.4)	3 (25.0)	0.436
PLECH score, n (%)				
<3	29 (48.3)	23 (47.9)	6 (50.0)	
≥3	31 (51.7)	25 (52.1)	6 (50.0)	1.00
PLECH score, (median [IQR])	3.00 [3.0]	3.00 [3.00]	3.00 [3.25]	0.4

ECOG, Eastern Cooperative Oncology Group. NRLN, non-regional lymph nodes. PLECH, Prognostic Factors in Pleural Mesothelioma Patients Receiving First-Line Chemotherapy including Platelet count (P: +2), high LDH (L: +1), ECOG ≥ 2 (E: +1), Chest pain at diagnosis (C: +2), and non-epithelioid Histology (H: +1). Trop-2, Trophoblast cell surface antigen-2. IHC, immunohistochemistry. IQR, interquartile range. **Multimodal therapy included chemotherapy, radiotherapy and surgery. ***Metastases sites may not sum 60 as each patient can have more than 1 metastatic site. Statistical differences were determined using chi-squared test or *Fisher exact test. Statistical significance was set at p < 0.05. Statistically significant p-values are highlighted in bold.

A significant proportion of patients experienced pleural effusion (40.0%) at diagnosis, followed by metastasis to contralateral pleura (30.0%), lung (26.7%), non-regional lymph nodes (NRLN) (21.7%), bone (18.3%), adrenal glands (3.3%), liver (3.3%), and brain (1.7%). Regarding first-line treatment, most patients (71.7%) received chemotherapy, while the rest underwent best supportive care (13.3%), multimodal therapy (chemotherapy, radiotherapy and surgery, 11.7%), and surgery only (3.3%) (**Table 1**). None of the patients in this study received ICIs in the first-line setting given that the approvals for the current standard-of-care immunotherapy regimens occurred toward the end of or after our enrollment window.

### 3.2 Baseline clinical features according to Trop-2 expression

**Figure 1:**
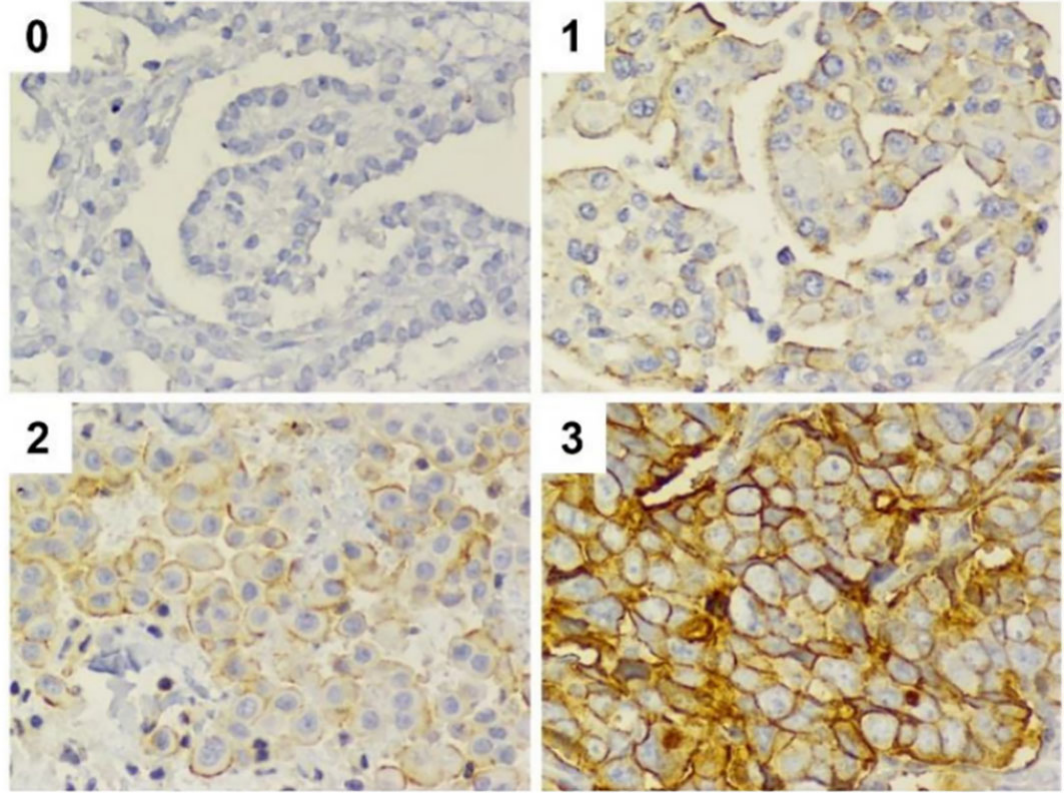
Representative immunohistochemical staining of Trop-2 in human pleural mesothelioma biopsies, demonstrating the staining intensity (0–3) used for further calculation of H-score values. Trop-2 expression was independently assessed by two pathologists specialized in oncology, who estimated both staining intensity and the percentage of stained tumor cells. The intensity of cell membrane staining was classified into four categories: no staining (0), weak (1+, light brown membrane staining), moderate (2+), and strong (3+, dark brown linear membrane staining). To ensure consistent scoring, strong staining (3+) was easily visible under a 4× objective lens, moderate staining (2+) required a 10× lens for clear visualization, and weak staining (1+) was distinguishable only at 40× magnification. The Histochemical Score (H-score) for each case was calculated by multiplying the staining intensity by the percentage of positive cells, yielding a total score ranging from 0 to 300.

As shown in **Figure 1**, Trop-2 positivity by IHC was observed in 12 (20.0%) of samples, with an inter-pathologist concordance of 100%. Most patients with positive Trop-2 expression harbored clinical stage III-IV (100.0% vs 66.7%, *p* = 0.049) and baseline metastases to lung (58.3% vs. 18.8%, *p* = 0.016) and NRLN (58.3% vs. 12.5%, *p* = 0.002) (**Table 1**).

### 3.3 Progression-free survival

**Figure 2:**
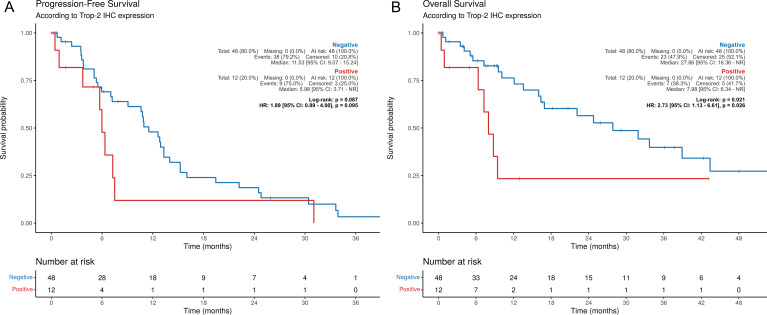
Kaplan-Meier curves of progression-free survival **(A)** or overall survival **(B)** stratified by Trop-2 expression in the whole cohort. HR, hazard ratio; CI, confidence interval; PFS, progression-free survival; OS, overall survival. TROP-2, Trophoblast cell surface antigen-2. IHC, immunohistochemistry. HR, hazard ratio. CI, confidence interval.

**Figure 3:**
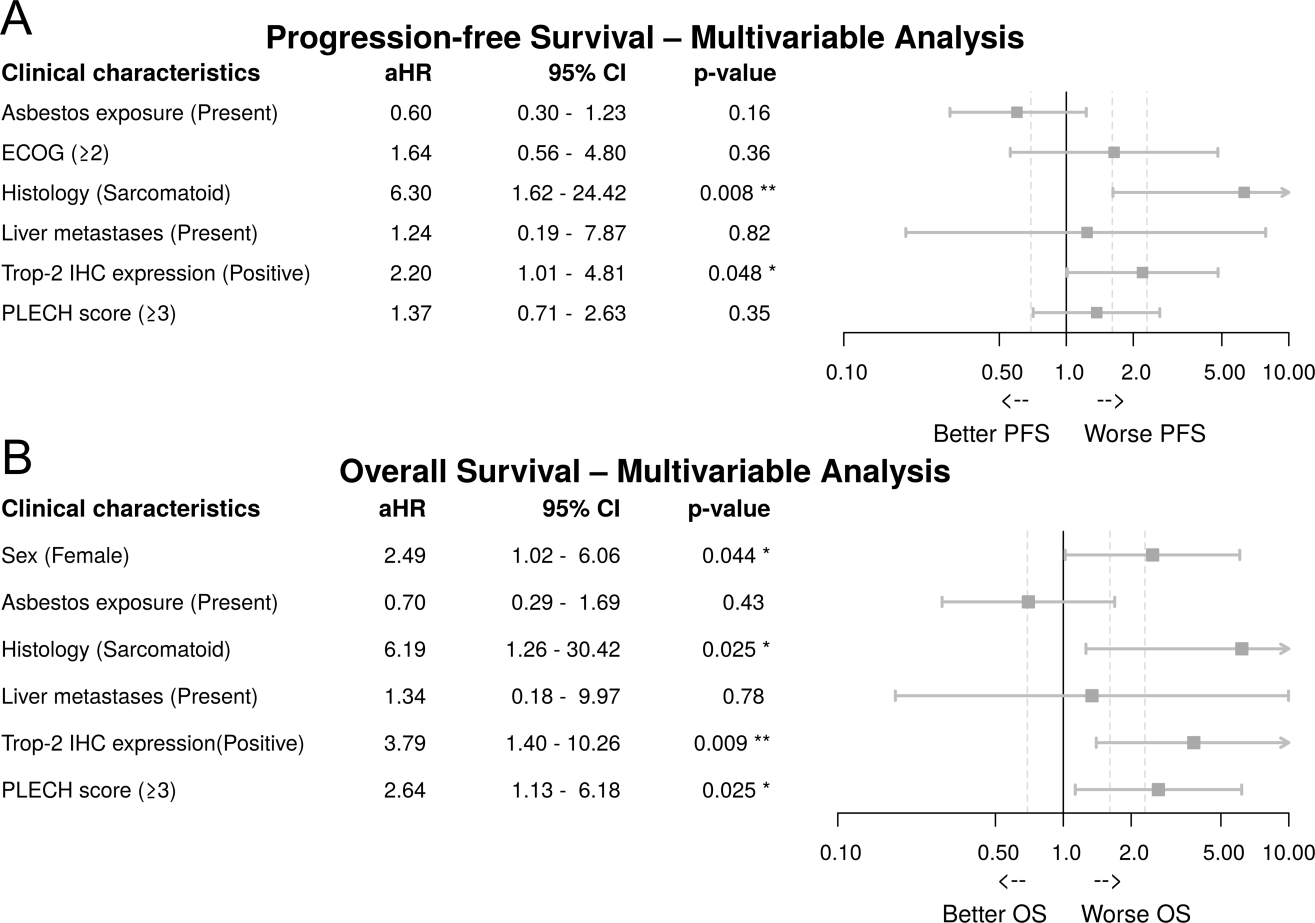
Clinical characteristics related to progression-free survival **(A)** and overall survival **(B)**. aHR, adjusted hazard ratio; CI, confidence interval; OS, overall survival; TROP-2, Trophoblast cell surface antigen-2; IHC, immunohistochemistry; ECOG PS, Eastern Cooperative Oncology Group Performance Status; PLECH, Prognostic Factors in Pleural Mesothelioma Patients Receiving First-Line Chemotherapy including Platelet count (P: +2), high LDH (L: +1), ECOG ≥ 2 (E: +1), Chest pain at diagnosis (C: +2), and non-epithelioid Histology (H: +1). mPFS, median progression-free survival. mOS, median overall survival.

After a median follow-up of 29.2 months (95% CI 16.6 - 48.0), the median PFS (mPFS) for the entire cohort was 10.8 months (95% CI 7.0-13.3). Shorter mPFS were displayed by patients with positive Trop-2 expression (5.9 vs. 11.5 months, *p* = 0.087; **Figure 2**), sarcomatoid histology (5.0 vs. 10.9 months, *p* < 0.001) and liver metastases (3.7 vs. 10.8 months, *p=*0.033) (**Table 2**). In multivariate analysis adjusted by asbestos exposure, ECOG PS, histological type, liver metastases, Trop-2 expression, and PLECH score, Trop-2 positivity (aHR 2.20, 1.0-4.8, *p* = 0.048) and sarcomatoid histology (aHR 6.30, 95% CI 1.6-24.2, *p* = 0.008) were independently related to PFS (**Figure 3**).

**Table 2:** Progression-free survival according to clinical characteristics.

	T,n	M,n	AR,n	E,n	C,n	Progression-free survival, months			Univariate analysis			Multivariate analysis		
Characteristics						Median	95% CI	p-value	uHR	95% CI	p-value	aHR	95% CI	p-value
All	60	0	60	47	13	10.8	7.03-13.3							
Sex, n														
Male	46	0	46	36	10	10.94	7.03-14.00	0.477	ref					
Female	14	0	14	11	3	7.26	3.81-NR		1.28	0.65-2.55	0.476			
Smoking status, n														
Non-smoker	28	0	28	21	7	7.49	5.72-19.45		ref					
Current/former	32	0	32	26	6	10.94	6.34-15.24	0.741	0.9	0.5-1.63	0.738			
Passive smoking, n														
Absent	50	0	50	40	10	9.07	6.34-12.91		ref					
Present	10	0	10	7	3	13.31	10.87-NR	0.318	0.66	0.29-1.49	0.322			
Wood smoke exposure, n														
Absent	43	0	43	32	11	10.87	7.49-15.24		ref					
Present	17	0	17	15	2	6.59	5.32-19.45	0.277	1.41	0.75-2.65	0.283			
Asbestos exposure, n														
Absent	39	0	39	32	7	10.61	6.34-13.31		ref					
Present	21	0	21	15	6	10.94	5.32-24.51	0.455	0.79	0.42-1.48	0.455	0.6	0.30-1.23	0.16
ECOG, n														
0-1	54	0	54	43	11	10.61	7.03-13.31		ref					
≥2	6	0	6	4	2	12.91	3.71-NR	0.694	1.24	0.44-3.51	0.682	1.64	0.56-4.80	0.36
Clinical stage, n														
I-II	16	0	16	13	3	13.31	10.78-NR		ref					
III-IV	44	0	44	34	10	7.26	5.95-12.62	0.586	1.2	0.62-2.31	0.59			
Histology, n														
Epithelioid	51	0	51	39	12	10.87	7.26-13.31		ref					
Biphasic	1	0	1	1	0	30.42	NR-NR		0.44	0.06-3.26	0.423			
Non-classified	3	0	3	2	1	30.36	3.81-NR		0.24	0.03-1.82	0.166			
Sarcomatoid	5	0	5	5	0	5.06	3.78-NR	**0.003**	4.93	1.76-13.81	**0.002**			
Sarcomatoid histology, n														
No sarcomatoid	55	0	55	42	13	10.94	7.49-14.00		ref					
Sarcomatoid	5	0	5	5	0	5.06	3.78-NR	**<0.001**	4.93	1.76-13.81	**0.002**	6.30	1.62-24.2	**0.008**
Contralateral pleural metastases, n														
Absent	42	0	42	32	10	10.78	7.2-14.0		ref					
Present	18	0	18	15	3	10.94	5.06-24.8	0.481	1.25	0.67-2.33	0.48			
Lung metastases, n														
Absent	44	0	44	34	10	10.78	6.34-14.00		ref					
Present	16	0	16	13	3	7.49	5.72-NR	0.935	0.97	0.51-1.86	0.936			
NRLN metastases, n														
Absent	47	0	47	38	9	10.78	6.34-13.31		ref					
Present	13	0	13	9	4	7.49	5.06-NR	0.504	0.78	0.37-1.63	0.508			
Brain metastases, n														
Absent	59	0	59	46	13	10.78	7.03-13.31		ref					
Present	1	0	1	1	0	7.2	NR-NR	0.502	1.96	0.26-14.64	0.512			
Adrenal metastases, n														
Absent	58	0	58	45	13	10.78	7.03-13.31		ref					
Present	2	0	2	2	0	16.72	2.4-NR	0.74	0.78	0.18-3.31	0.737			
Bone metastases, n														
Absent	49	0	49	38	11	10.94	7.26-15.24		ref					
Present	11	0	11	9	2	5.06	3.71-NR	0.116	1.79	0.86-3.77	0.122			
Liver metastases, n														
Absent	58	0	58	45	13	10.87	7.2-13.31		ref					
Present	2	0	2	2	0	3.73	0.43-NR	**0.033**	4.29	0.99-18.55	0.051	1.24	0.19-7.87	0.82
First-line treatment, n														
Chemotherapy only	43	0	43	38	5	7.49	5.98-12.81		ref					
Multimodal therapy*	7	0	7	5	2	15.64	15.24-NR		0.45	0.17-1.14	0.091			
Surgery only	2	0	2	0	2		NR-NR		NR	NR-NR	0.997			
Best supportive care	8	0	8	4	4	28.91	0.69-NR	0.309	0.76	0.22-2.57	0.656			
Trop-2 IHC expression														
Negative	48	0	48	38	10	11.53	9.07-15.24		ref					
Positive	12	0	12	9	3	5.98	3.71-NR	0.087	1.89	0.89-4.0	0.095	2.20	1.01-4.81	**0.048**
PLECH score, n														
<3	29	0	29	23	6	12.81	10.61-19.45		ref					
≥ 3	31	0	31	24	7	7.26	5.32-12.91	0.298	1.35	0.76-2.43	0.307	1.37	0.71-2.63	0.35

mPFS, median progression-free survival. ECOG, Eastern Cooperative Oncology Group. NRLN, non-regional lymph nodes. T, Total cases. M, Missing cases. AR, cases at risk. E, Events. C, censored cases. PLECH, Prognostic Factors in Pleural Mesothelioma Patients Receiving First-Line Chemotherapy including Platelet count (P: +2), high LDH (L: +1), ECOG ≥ 2 (E: +1), Chest pain at diagnosis (C: +2), and non-epithelioid Histology (H: +1). TROP-2, Trophoblast cell surface antigen-2. IHC, immunohistochemistry. aHR, adjusted hazard ratio. NR, not reached. *Multimodal therapy included chemotherapy, radiotherapy and surgery. Statistical significance was set at p < 0.05. Statistically significant p-values are highlighted in bold.

Among individuals treated with first-line chemotherapy (n=43), shorter mPFS was observed in those with sarcomatoid histology (5.1 vs. 10.6 months, *p* = 0.015) or Trop-2 expression (6.1 vs. 10.7 months, *p* = 0.044). In multivariate analysis adjusted by histological type, Trop-2 expression, and PLECH score, Trop-2 positivity (aHR 3.20, 95%CI 1.12-9.15, *p* = 0.029) and sarcomatoid histology (aHR 4.54, 95%CI 1.24-16.56, *p* = 0.021) were independently related to PFS (**Supplementary Table 2**).

### 3.4 Overall survival

The mOS for the entire cohort was 22.2 months (95% CI 13.6-43.5). A shorter mOS was displayed by patients with positive Trop-2 expression (7.9 vs. 27.8 months, *p* = 0.021, **Figure 2**), sarcomatoid histology (7.2 vs. 27.8 months, *p* = 0.001), liver metastases (5.2 vs. 24.7, *p* = 0.014), and PLECH score >3 (12.0 vs. 33.7 months, *p* = 0.034) (**Table 3**). In multivariate analysis adjusted by sex, asbestos exposure, histological type, liver metastases, Trop-2 expression and PLECH score, female sex (aHR 2.49, 95%CI 1.02-6.06, *p* = 0.044), sarcomatoid histology (aHR 6.19, 95% CI 1.26-30.42, *p* = 0.025), Trop-2-positive disease (aHR 3.79, 95% CI 1.40-10.26, *p* = 0.009), and PLECH score >3 (aHR 2.64, 95% CI 1.13-6.18, *p* = 0.025) were independently associated with worse OS (**Figure 3**).

**Table 3:** Overall survival according to clinical characteristics.

Characteristics	T,n	M,n	AR,n	E,n	C,n	Overall survival, months			Univariate analysis			Multivariate analysis		
						Median	95% CI	p-value	uHR	95% CI	p-value	aHR	95% CI	p-value
All	60	0	60	30	30	22.2	13.6-43.4							
Sex, n														
Male	46	0	46	22	24	27.86	16.0 - NR		ref					
Female	14	0	14	8	6	8.77	5.06 - NR	0.1	1.97	0.87-4.5	0.106	2.49	1.02-6.06	**0.044**
Smoking status, n														
Non-smoker	28	0	28	16	12	12.09	8.77 - NR		ref					
Current/former	32	0	32	14	18	33.71	16.0 - NR	0.082	0.52	0.24 - 1.1	0.086			
Passive smoking, n														
Absent	50	0	50	27	23	22.14	10.12 - 38.93		ref					
Present	10	0	10	3	7	116.93	16.36 - NR	0.066	0.28	0.07 - 1.19	0.084			
Wood smoke exposure, n														
Absent	43	0	43	24	19	16.92	10.12 - 43.4		ref					
Present	17	0	17	6	11	38.93	24.77 - NR	0.25	0.59	0.24 - 1.46	0.256			
Asbestos exposure, n														
Absent	39	0	39	20	19	22.14	12.09 - NR		ref					
Present	21	0	21	10	11	31.9	10.12 - NR	0.998	1	0.46 - 2.16	1	0.70	0.29-1.69	0.43
ECOG, n														
0-1	54	0	54	28	26	22.14	12.09 - 43.4		ref					
≥2	6	0	6	2	4	16.92	16.92 - NR	0.763	0.8	0.19 - 3.39	0.763			
Clinical stage, n														
I-II	16	0	16	8	8	27.86	16.0 - NR		ref					
III-IV	44	0	44	22	22	22.14	9.43 - NR	0.778	1.13	0.49 - 2.58	0.776			
Histology, n														
Epithelioid	51	0	51	24	27	24.77	16.0 - NR		ref					
Biphasic	1	0	1	1	0	38.93	NR - NR		0.89	0.12 - 6.63	0.906			
Non-classified	3	0	3	1	2	56.9	NR - NR		0.44	0.05 - 3.59	0.446			
Sarcomatoid	5	0	5	4	1	7.26	4.11 - NR	**0.012**	5.54	1.75 - 17.51	**0.004**			
Sarcomatoid histology, n														
No sarcomatoid	55	0	55	26	29	27.86	16.36 - NR		ref					
Sarcomatoid	5	0	5	4	1	7.26	4.11 - NR	**0.001**	5.54	1.75 - 17.51	**0.004**	6.19	1.26-30.42	**0.025**
Contralateral Pleural metastases, n														
Absent	42	0	42	19	23	16.92	12.09 - NR		ref					
Present	18	0	18	11	7	24.77	7.26 - NR	0.962	1.02	0.47 - 2.21	0.96			
Lung metastases, n														
Absent	44	0	44	23	21	22.14	13.57 - NR		ref					
Present	16	0	16	7	9	116.93	8.77 - NR	0.25	0.59	0.24 - 1.47	0.258			
NRLN metastases, n														
Absent	47	0	47	25	22	22.14	13.57 - NR		ref					
Present	13	0	13	5	8	31.9	7.26 - NR	0.814	0.89	0.34 - 2.35	0.817			
Brain metastases, n														
Absent	59	0	59	30	29	22.14	13.57 - 43.4		ref					
Present	1	0	1	0	1		NR - NR	0.556	NR	NR-NR	0.998			
Adrenal metastases, n														
Absent	58	0	58	30	28	22.14	12.09 - 43.4		ref					
Present	2	0	2	0	2		NR - NR	0.228	NR	NR-NR	0.998			
Bone metastases, n														
Absent	49	0	49	23	26	27.86	16.36 - NR		ref					
Present	11	0	11	7	4	9.56	4.11 - NR	0.062	2.23	0.94 - 5.31	0.069			
Liver metastases, n														
Absent	58	0	58	28	30	24.77	16.0 - NR		ref					
Present	2	0	2	2	0	5.27	0.43 - NR	**0.014**	5.23	1.18 - 23.1	**0.029**	1.34	0.18-9.97	0.78
First-line treatment, n														
Chemotherapy only	43	0	43	23	20	16.36	10.12 - NR		ref					
Multimodal therapy*	7	0	7	3	4	27.86	22.14 - NR		0.58	0.17 - 1.94	0.375			
Surgery only	2	0	2	0	2		NR - NR		NR	NR-NR	0.998			
Best supportive care	8	0	8	4	4	56.9	0.69 - NR	0.445	2.25	0.72 - 7	0.16			
Trop-2 IHC expression														
Negative	48	0	48	23	25	27.86	16.36 - NR		ref					
Positive	12	0	12	7	5	7.98	6.34 - NR	**0.021**	2.73	1.13 - 6.61	**0.026**	3.79	1.40-10.26	**0.009**
PLECH score, n														
<3	29	0	29	12	17	33.71	16.36 - NR		ref					
≥ 3	31	0	31	18	13	12.09	7.26 - NR	**0.034**	2.24	1.05 - 4.79	**0.038**	2.64	1.13-6.18	**0.025**

mOS, median overall survival. ECOG PS, Eastern Cooperative Oncology Group Performance Status. NRLN, non-regional lymph nodes. T, Total cases. M, Missing cases. AR, cases at risk. E, Events. C, censored cases. *Multimodal therapy included chemotherapy, radiotherapy and surgery. PLECH, Prognostic Factors in Pleural Mesothelioma Patients Receiving First-Line Chemotherapy including Platelet count (P: +2), high LDH (L: +1), ECOG ≥ 2 (E: +1), Chest pain at diagnosis (C: +2), and non-epithelioid Histology (H: +1). Trop-2, Trophoblast cell surface antigen-2. IHC, immunohistochemistry. aHR, adjusted hazard ratio. NR, not reached. Statistical significance was set at p < 0.05. Statistically significant p-values are highlighted in bold.

Among patients undergoing first-line chemotherapy (n=43), a shorter mOS was experienced by those with sarcomatoid histology (7.27 vs. 24.7 months, *p* = 0.016) or Trop-2 expression (8.38 vs. 31.9 months, *p* = 0.011). In multivariate analysis adjusted by histological type, trop-2 expression and PLECH score, sarcomatoid histology (HR 4.86, 95%CI 1.11-21.16, *p* = 0.034) and Trop-2 expression (HR 6.07, 95%CI 1.73-21.24, *p=*0.004) were independently related to OS (**Supplementary Table 3**).

### 3.5 Therapeutic response according to Trop-2 expression

As shown in **Supplementary Figures 3** and **4**, no statistically significant differences in objective response rates (ORR) to first-line treatment were observed based on Trop-2 positivity, either within the whole cohort (44.4% vs. 50%; p = 1.00) or among patients treated with chemotherapy (28.6% vs. 50.0%; p = 0.418).

## 4 Discussion

In this study, Trop-2 was expressed in 20.0% of cases, exclusively in those with epithelioid histology and late-stage disease and represented an independent predictor of worse PFS and OS. This frequency is higher than that reported in Caucasian populations (15.6%), but lower than pleural effusion-derived PM cell lines (35%) (15). This suggests that ethnicity and methodology confer a wide variability for Trop-2 expression across studies (13.6%-73.0%) (15, 27–29). Notably, none of the non-epithelioid PM cases in our study expressed Trop-2, contrasting with the 15.4% positivity reported by Dum et al. (30) This discrepancy may arise from differences in genetic background, specificity of detection antibodies, sample type (tissue sections vs. microarrays), and immunohistochemical staining positivity criteria. Thus, these findings highlight the need to standardize Trop-2 assessment methodologies in PM.

Currently, there is no consensus on standardized interpretation criteria for Trop-2 immunohistochemistry across tumor types. The most widely adopted quantitative method to determine Trop-2 positivity relies on H-score positivity, derived from the product of staining membrane intensity and the percentage of stained cells, generally employed in breast and urothelial cancer studies (24, 31). As Trop-2 is relatively prevalent in these malignancies (typically 78–90%), its expression is often categorized into low (0 to 100), medium (100–200), and high (>200). Accordingly, we determined Trop-2 positivity based on positive H-score values as a continuous variable (range 1–300), but avoided the performance of cutoff dichotomizations, as this would result in small and statistically unmeaningful subgroups for later analysis. Despite this difference, our findings are driven by cases with substantial TROP2 protein density; 91.6% (11/12) of positive cases exhibited moderate (2+) to strong (3+) intensity, 75% (9/12) showed expression in ≥80% of tumor cells, and the median H-score among positive cases was 240 (range 60–300) (**Supplementary Table 1**). Therefore, the prognostic associations observed in our study reflect biologically meaningful TROP2 expression rather than trace or borderline staining. Furthermore, our approach is consistent with studies evaluating Trop-2 in other rare tumors, including sebaceous carcinoma (n=14), sweat gland carcinoma (n=18) (32), cutaneous squamous cell carcinomas (SCCs; n = 44), basal cell carcinomas (BCCs; n = 27) and Merkel cell carcinomas (MCCs; n = 12), melanomas (n = 25) (27), which also reported Trop-2 positivity as by using H-score as a continuous variable without a predefined cutoff.

Moreover, our study did not identify significant associations between Trop-2 expression and environmental exposures, including tobacco use and asbestos exposure. Although *in vitro* evidence has demonstrated that cigarette smoke extract (CSE) upregulates Trop-2 expression in airway basal cells through p38 MAPK and NF-κB pathway activation in a dose- and time-dependent manner, this relationship was established exclusively in airway basal cells, which are histogenetically distinct from mesothelial cells (33). As well, clinical data has suggested that PM is not related to cigarette smoke as this may not reach the mesothelium (34). Moreover, Trop-2 is not expressed in normal mesothelial cells, suggesting that Trop-2 upregulation in mesothelioma is an event intrinsic to tumor biology rather than a direct consequence of environmental carcinogen exposure (15).

Notably, Trop-2 was independently associated with poor survival in our cohort, which may derive from its biological role; Trop-2 overexpression in murine models of PM promotes pro-oncogenic, stemness-associated, and metastasis-related transcriptional programs (29). This might also explain the association between Trop-2 and late-stage disease, lung metastatic involvement, and NRLN metastases. However, its prognostic impact appears to be tumor-specific, potentially derived from differential roles across distinct cancer types (17). For instance, Trop-2 predicts poor survival in lung adenocarcinoma, whereas it does not modify the prognosis of squamous cell carcinomas. Nonetheless, its deleterious role in PM identifies it as a high-value therapeutic vulnerability. Consequently, this deleterious impact establishes a strong rationale for the clinical implementation of Trop-2-directed ADCs, as targeting such a potent driver of progression may yield transformative results similar to those observed with datopotamab deruxtecan in NSCLC (35). In this regard, a phase II clinical trial (NCT06477419) is currently underway to evaluate the overall response rate of sacituzumab govitecan in patients with diffuse PM after progression on platinum/pemetrexed and/or immunotherapy (36).

Importantly, the benefit of characterizing Trop-2 expression in PM extends beyond its role as an ADC target; it may also refine patient selection for ICIs. While we acknowledge that none of the patients in the present cohort received immunotherapy, limiting our ability to provide direct clinical correlation, the hypothesis that Trop-2 may serve as a surrogate marker of resistance is supported by mechanistic evidence linking its expression to diminished T-cell infiltration and the suppression of apoptosis (37). Consequently, approximately one in four patients with PM expressing Trop-2 may exhibit diminished sensitivity to this therapeutic alternative. This observation may be relevant as ICI combinations, such as nivolumab plus ipilimumab, are the preferred first-line treatment for non-epithelioid PM, a subtype in which Trop-2 expression is rare, potentially explaining the greater therapeutic benefit observed in this group (9). However, in patients with epithelioid histology unable for chemotherapy, Trop-2 expression could be a negative predictor for ICIs, which represent acceptable treatment options in this subgroup. Consequently, Trop-2 identification may be relevant for selecting patients with potential benefit from chemoimmunotherapy or alternative therapeutic strategies other than ICIs.

Beyond Trop-2 expression, our multivariable analysis also identified other independent predictors of mortality in this population. Sarcomatoid histology and a PLECH score >3 were strongly associated with worse OS, underscoring the high biological risk related to these features. The independent association of female sex with worse OS is unexpected, as existing literature generally identifies it as a protective factor (38). In our cohort, this finding may be explained by a cumulative burden of high-risk features within this small female subgroup (n=14), as detailed in **Supplementary Table 4**. Specifically, women exhibited a notably higher proportion of Trop-2 positivity compared to men (35.7% vs. 15.2%, p=0.195). Given that Trop-2 was the strongest independent predictor of mortality in our study, its higher prevalence in women likely acts as a significant biological confounder.

Moreover, a higher percentage of women presented with clinical stage III–IV (85.7% vs. 69.6%) and pleural effusion (57.1% vs. 34.8%), suggesting a higher disease burden at diagnosis. Lastly, while asbestos exposure was comparable between sexes (35.7% in females vs. 34.8% in males, p=1.000), women faced a trend to higher additional burden of passive smoking (35.7% vs. 10.9%, p=0.076). Of note, female sex was not significantly associated with OS in the univariate analysis; its emergence as a significant factor only after multivariate adjustment suggests it serves as a proxy for the clustering of the aforementioned adverse clinical features. Consequently, we explicitly caution against overinterpreting this finding as a generalizable biological trend, as it likely reflects the specific interplay of environmental and molecular risks in our population.

A relatively low rate of documented asbestos exposure was found in our cohort (35%) compared to the higher prevalence reported in European and Asian series, which often ranges from 62% to 92% (39–41). This discrepancy is a notable finding that warrants careful interpretation. First, we acknowledge the inherent potential for underreporting or recall bias; patients are frequently unaware of indirect, domestic, or para-occupational asbestos contact, which may lead to an underestimation of historical exposure.

Furthermore, this prevalence may be influenced by referral bias. As a public tertiary center receiving patients from diverse regions, our cohort likely includes individuals with environmental or non-industrial exposures that are harder to quantify than traditional occupational roles. Notably, our data are consistent with previous reports from other Mexican studies (42–44), suggesting that the clinical landscape of MPM in our region frequently involves patients without a clear history of industrial contact. This pattern may point toward different environmental triggers or low-intensity chronic exposures unique to our population, highlighting the need for broader epidemiological screening tools in developing countries.

The primary strength of this study lies in the characterization of Trop-2 expression frequency in a real-world cohort of patients with PM, alongside its clinical correlates and prognostic significance. The main limitation of this study is the relatively small sample size (N = 60), with only 12 patients (20%) exhibiting TROP-2 positivity, which limits the statistical power of subgroup analyses. However, this sample size is consistent with the rare incidence of this disease, widely recognized by international guidelines as an intrinsic limitation in the study of pleural mesothelioma (45). Despite this, our study benefits from perfect inter-observer concordance (100%) in TROP-2 assessment, multivariable Cox regression adjusting for established prognostic factors, a prolonged median follow-up of 29.2 months, and biologically coherent associations between TROP-2 positivity and metastatic disease. Nevertheless, these findings should be considered hypothesis-generating and require validation by prospective multicenter cohorts. Notably, other practice-influencing studies in pleural mesothelioma have been conducted with comparable or smaller cohorts, including retrospective or prospective observational designs (46–49).

Another limitation of this study is the absence of immunotherapy outcome data, as none of the patients in our cohort received ICIs. This reflects the treatment landscape during our inclusion period (2010–2022). Although US FDA approved nivolumab plus ipilimumab based on the CheckMate 743 trial on October 2020 (50), this therapeutic approach was not immediately available in our setting, which first published experience with nivolumab plus ipilimumab was published on 2024, including patients treated predominantly after the FDA approval (12).

## 5 Conclusion

This study establishes that Trop-2 expression defines a clinically relevant PM subgroup, providing a compelling rationale for biomarker-driven trials of Trop-2-targeted therapies.

## Data Availability

The original contributions presented in the study are included in the article/**Supplementary Material**, further inquiries can be directed to the corresponding author/s.

## References

[1] Bou-Samra P Chang A Azari F Kennedy G Segil A Guo E . Epidemiological, therapeutic, and survival trends in Malignant pleural mesothelioma: a review of the National Cancer Database. Cancer Med. (2023) 12:12208–20. doi: 10.1002/cam4.5915. PMID: 37062067 PMC10278474

[2] Huang J Chan SC Pang WS Chow SH Lok V Zhang L . Global incidence, risk factors, and temporal trends of mesothelioma: a population-based study. J Thorac Oncol Off Publ Int Assoc Study Lung Cancer. (2023) 18:792–802. doi: 10.1016/j.jtho.2023.01.095. PMID: 36775192

[3] Gilham C Rake C Hodgson J Darnton A Burdett G Peto Wild J . Past and current asbestos exposure and future mesothelioma risks in Britain: the Inhaled Particles Study (TIPS). Int J Epidemiol. (2018) 47:1745–56. doi: 10.1093/ije/dyx276. PMID: 29534192 PMC6280925

[4] Avilés-Salas A Cabrera-Miranda L Hernández-Pedro N Vargas-Lías DS Samtani S Muñoz-Montaño W . PD-L1 expression complements CALGB prognostic scoring system in Malignant pleural mesothelioma. Front Oncol. (2023) 13:1269029. doi: 10.3389/fonc.2023.1269029. PMID: 38111532 PMC10725960

[5] Rojas L Cardona AF Trejo-Rosales R Zatarain-Barrón ZL Ramírez-Tirado L-A Ruiz-Patiño A . Characteristics and long-term outcomes of advanced pleural mesothelioma in Latin America (MeSO-CLICaP). Thorac Cancer. (2019) 10:508–18. doi: 10.1111/1759-7714.12967. PMID: 30706690 PMC6397921

[6] van Kooten JP Belderbos RA von der Thüsen JH Aarts MJ Verhoef C Burgers JA . Incidence, treatment and survival of Malignant pleural and peritoneal mesothelioma: a population-based study. Thorax. (2022) 77:1260–7. doi: 10.1136/thoraxjnl-2021-217709. PMID: 35149582 PMC9685713

[7] Husain AN Colby TV Ordóñez NG Allen TC Attanoos RL Beasley MB . Guidelines for pathologic diagnosis of Malignant mesothelioma 2017 update of the consensus statement from the International Mesothelioma Interest Group. Arch Pathol Lab Med. (2018) 142:89–108. doi: 10.5858/arpa.2017-0124-RA. PMID: 28686500

[8] Dacic S . Pleural mesothelioma classification-update and challenges. Mod Pathol Off J U S Can Acad Pathol Inc. (2022) 35:51–6. doi: 10.1038/s41379-021-00895-7. PMID: 34465883

[9] Baas P Scherpereel A Nowak AK Fujimoto N Peters S Tsao AS . First-line nivolumab plus ipilimumab in unresectable Malignant pleural mesothelioma (CheckMate 743): a multicentre, randomised, open-label, phase 3 trial. Lancet Lond Engl. (2021) 397:375–86. doi: 10.1016/S0140-6736(20)32714-8. PMID: 33485464

[10] Chu Q Perrone F Greillier L Tu W Piccirillo MC Grosso F . Pembrolizumab plus chemotherapy versus chemotherapy in untreated advanced pleural mesothelioma in Canada, Italy, and France: a phase 3, open-label, randomised controlled trial. Lancet Lond Engl. (2023) 402:2295–306. doi: 10.1016/S0140-6736(23)01613-6. PMID: 37931632

[11] de Miguel-Perez D Pickering EM Malapelle U Grier W Pepe F Pisapia P . Genomic profiling of tissue and blood predicts survival outcomes in patients with resected pleural mesothelioma. Eur J Cancer Oxf Engl 1990. (2024) 196:113457. doi: 10.1016/j.ejca.2023.113457. PMID: 38008032

[12] Enrico D Gomez JE Aguirre D Tissera NS Tsou F Pupareli C . Efficacy of first-line nivolumab plus ipilimumab in unresectable pleural mesothelioma: a multicenter real-world study (ImmunoMeso LATAM). Clin Lung Cancer. (2024) 25:723–731.e2. doi: 10.1016/j.cllc.2024.09.005. PMID: 39424512

[13] Qiu S Zhang J Wang Z Lan H Hou J Zhang N . Targeting Trop-2 in cancer: recent research progress and clinical application. Biochim Biophys Acta Rev Cancer. (2023) 1878:188902. doi: 10.1016/j.bbcan.2023.188902. PMID: 37121444

[14] Lombardi P Filetti M Falcone R Altamura V Paroni Sterbini F Bria E . Overview of Trop-2 in cancer: from pre-clinical studies to future directions in clinical settings. Cancers. (2023) 15:1744. doi: 10.3390/cancers15061744. PMID: 36980630 PMC10046386

[15] Hegedüs L Okumus Ö Mairinger F Ploenes T Reuter S Schuler M . TROP2 expression and SN38 antitumor activity in Malignant pleural mesothelioma cells provide a rationale for antibody-drug conjugate therapy. Lung Cancer. (2023) 178:237–46. doi: 10.1016/j.lungcan.2023.03.003. PMID: 36907051

[16] Zhang X Xiao H Na F Sun J Guan Q Liu R . Evolution of TROP2: biological insights and clinical applications. Eur J Med Chem. (2025) 296:117863. doi: 10.1016/j.ejmech.2025.117863. PMID: 40517574

[17] Inamura K Yokouchi Y Kobayashi M Ninomiya H Sakakibara R Subat S . Association of tumor TROP2 expression with prognosis varies among lung cancer subtypes. Oncotarget. (2017) 8:28725–35. doi: 10.18632/oncotarget.15647. PMID: 28404926 PMC5438686

[18] Bardia A Hurvitz SA Tolaney SM Loirat D Punie K Oliveira M . Sacituzumab govitecan in metastatic triple-negative breast cancer. N Engl J Med. (2021) 384:1529–41. doi: 10.1056/NEJMoa2028485. PMID: 33882206

[19] Rugo HS Bardia A Marmé F Cortés J Schmid P Loirat D . Overall survival with sacituzumab govitecan in hormone receptor-positive and human epidermal growth factor receptor 2-negative metastatic breast cancer (TROPiCS-02): a randomised, open-label, multicentre, phase 3 trial. Lancet. (2023) 402:1423–33. doi: 10.1016/S0140-6736(23)01245-X. PMID: 37633306

[20] Ahn M-J Tanaka K Paz-Ares L Cornelissen R Girard N Pons-Tostivint E . Datopotamab deruxtecan versus docetaxel for previously treated advanced or metastatic non-small cell lung cancer: the randomized, open-label phase III TROPION-Lung01 study. J Clin Oncol Off J Am Soc Clin Oncol. (2025) 43:260–72. doi: 10.1200/JCO-24-01544. PMID: 39250535 PMC11771353

[21] Koltai T Fliegel L . The relationship between Trop-2, chemotherapeutic drugs, and chemoresistance. Int J Mol Sci. (2023) 25:87. doi: 10.3390/ijms25010087. PMID: 38203255 PMC10779383

[22] Italiano A Leroy L Guegan J-P Cousin S Peyraud F Cabart M . TROP2 expression and response to immune checkpoint inhibition in patients with advanced non-small cell lung cancer. J Clin Oncol. (2023) 41:9040. doi: 10.1200/JCO.2023.41.16_suppl.9040. PMID: 41909186

[23] Guijosa A Cabrera-Miranda LA Gómez-García AP Trejo Rosales R Muñoz-Montaño W Flores D . Prognostic factors in pleural mesothelioma patients receiving first-line chemotherapy: establishing the PLECH baseline risk score. Oncology. (2025) 104:1–16. doi: 10.1159/000543637. PMID: 40068665 PMC12668723

[24] Rojo Todo FG Lopez-Tarruella Cobo S Barrios CH Torrecillas Torres L Ruiz M Bines J . TROP2 overexpression as predictor of outcome in patients with early triple-negative breast cancer. Exploratory analysis from the GEICAM_CIBOMA trial. J Clin Oncol. (2025) 43:582. doi: 10.1200/JCO.2025.43.16_suppl.582

[25] R Core Team . R: a language and environment for statistical computing. In: R: a language and environment for statistical computing (2025). Available online at: https://www.R-project.org/.

[26] Therneau TM . A package for survival analysis in R (2024). Available online at: https://CRAN.R-project.org/package=survival (Accessed September 1, 2025).

[27] He LJ Wang Y Curry JL Ning J Aung PP Nagarajan P . TROP2 is highly expressed in cutaneous squamous cell carcinomas and a subset of adnexal carcinomas: a potential therapeutic target with TROP2-directed antibody drug conjugates. Hum Pathol. (2025) 160:105853. doi: 10.1016/j.humpath.2025.105853. PMID: 40523445

[28] Menz A Lony N Lennartz M Dwertmann Rico S Schlichter R Kind S . Epithelial cell adhesion molecule (EpCAM) expression in human tumors: a comparison with pan-cytokeratin and TROP2 in 14,832 tumors. Diagn Basel Switz. (2024) 14:1044. doi: 10.3390/diagnostics14101044. PMID: 38786342 PMC11120328

[29] Offin MD Tischfield S Manoj P Agrawal P Tendler S Pratt EC . MA05.04 Targeting TROP-2 in diffuse pleural mesothelioma. J Thorac Oncol. (2024) 19:S69. doi: 10.1016/j.jtho.2024.09.125. PMID: 38826717

[30] Dum D Taherpour N Menz A Höflmayer D Völkel C Hinsch A . Trophoblast cell surface antigen 2 expression in human tumors: a tissue microarray study on 18,563 tumors. Pathobiol J Immunopathol Mol Cell Biol. (2022) 89:245–58. doi: 10.1159/000522206. PMID: 35477165 PMC9393818

[31] Yang J Zhang F Cai X Ding Z Xie C Qiu H . Trophoblast cell-surface antigen 2 expression in digestive neoplasms: a promising target for antibody-drug conjugates. Oncologist. (2025) 30:oyaf320. doi: 10.1093/oncolo/oyaf320. PMID: 41100061 PMC12659696

[32] Ito T Hashimoto H Tanaka Y Tanegashima K Murata M Ichiki T . TROP2 expression in sebaceous and sweat gland carcinoma. J Clin Med. (2022) 11:607. doi: 10.3390/jcm11030607. PMID: 35160059 PMC8836355

[33] Li H Cui L Liu Q Dou S Wang W Xie M . Ginsenoside Rb3 alleviates CSE-induced TROP2 upregulation through p38 MAPK and NF-κB pathways in basal cells. Am J Respir Cell Mol Biol. (2021) 64:747–59. doi: 10.1165/rcmb.2020-0208OC. PMID: 33705682

[34] Robinson BW Musk AW Lake RA . Malignant mesothelioma. Lancet. (2005) 9483:397–408. doi: 10.1016/S0140-6736(05)67025-0. PMID: 16054941

[35] Garassino MC Sands J Paz-Ares L Lisberg A Johnson M Pérol M . PL02.11 Normalized membrane ratio of TROP2 by quantitative continuous scoring is predictive of clinical outcomes in TROPION-Lung 01. J Thorac Oncol. (2024) 19:S2–3. doi: 10.1016/j.jtho.2024.09.015. PMID: 38826717

[36] Memorial Sloan Kettering Cancer Center Phase 2 study of sacituzumab govitecan-hziy in patients with previously treated mesothelioma (2025). Available online at: https://clinicaltrials.gov/study/NCT06477419 (Accessed July 10, 2025).

[37] Bessede A Peyraud F Besse B Cousin S Cabart M Chomy F . TROP2 is associated with primary resistance to immune checkpoint inhibition in patients with advanced non-small cell lung cancer. Clin Cancer Res Off J Am Assoc Cancer Res. (2024) 30:779–85. doi: 10.1158/1078-0432.CCR-23-2566. PMID: 38048058 PMC10870116

[38] Taioli E Wolf AS Camacho-Rivera M Flores RM . Women with Malignant pleural mesothelioma have a threefold better survival rate than men. Ann Thorac Surg. (2014) 98:1020–4. doi: 10.1016/j.athoracsur.2014.04.040. PMID: 24928677

[39] Burdorf A Järvholm B Englund A . Explaining differences in incidence rates of pleural mesothelioma between Sweden and the Netherlands. Int J Cancer. (2005) 113:298–301. doi: 10.1002/ijc.20552. PMID: 15386418

[40] Yates DH Corrin B Stidolph PN Browne K . Malignant mesothelioma in south east England: clinicopathological experience of 272 cases [published erratum appears in Thorax 1997 Nov;52(11):1018. Thorax. (1997) 52:507–12. doi: 10.1136/thx.52.6.507. PMID: 9227715 PMC1758573

[41] Albin M Magnani C Krstev S Rapiti E Shefer I . Asbestos and cancer: an overview of current trends in Europe. Environ Health Perspect. (1999) 107:289–98. doi: 10.1289/ehp.99107s2289. PMID: 10350513 PMC1566265

[42] Ortega-Guerrero MA Carrasco-Núñez G Barragán-Campos H Ortega MR . High incidence of lung cancer and Malignant mesothelioma linked to erionite fibre exposure in a rural community in Central Mexico. Occup Environ Med. (2015) 72:216–8. doi: 10.1136/oemed-2013-101957. PMID: 25231672

[43] Avilés-Salas A Dupont DD Aranda-Gutierrez A Heredia-Jara AN Caballé-Perez E Cabrera-Miranda L . Clinical, diagnostic and prognostic relevance of GATA3 in Malignant pleural mesothelioma: a retrospective cohort study. BMC Cancer. (2026) 26:436. doi: 10.1186/s12885-026-15692-1. PMID: 41928138 PMC13054999

[44] García-López M Barrera-Rodríguez R . Mesotelioma Maligno: descripción clínica y radiológica de 45 casos con y sin exposición a asbestos. Salud Pública México. (2000) 42:511–9. doi: 10.1590/S0036-36342000000600007. PMID: 11201579

[45] Kindler HL Ismaila N Bazhenova L Chu Q Churpek JE Dagogo-Jack I . Treatment of pleural mesothelioma: ASCO guideline update. J Clin Oncol. (2025) 43:1006–38. doi: 10.1200/JCO-24-02425. PMID: 39778125

[46] Herrera M Ucero ÁC Enguita AB Carrizo N Yarza R Gómez-Randulfe I . PD-L1, VISTA, and CD47 expression and prognosis impact in Malignant pleural mesothelioma. J Clin Oncol. (2022) 40:8562. doi: 10.1200/JCO.2022.40.16_suppl.8562. PMID: 41909186

[47] Chapel DB Hornick JL Barlow J Bueno R Sholl LM . Clinical and molecular validation of BAP1, MTAP, P53, and Merlin immunohistochemistry in diagnosis of pleural mesothelioma. Mod Pathol. (2022) 35:1383–97. doi: 10.1038/s41379-022-01081-z. PMID: 35459788 PMC9529776

[48] Bizzarri FP Campetella M Russo P Palermo G Moosavi SK Rossi F . Prognostic value of PLR, SIRI, PIV, SII, and NLR in non-muscle invasive bladder cancer: can inflammatory factors influence pathogenesis and outcomes? Cancers. (2025) 17:2189. doi: 10.3390/cancers17132189. PMID: 40647487 PMC12248968

[49] Sharova E Del Bianco P Urso L Silic-Benussi M Scattolin D D’Agostino DM . Circulating microRNAs as biomarkers for risk assessment and prognostic stratification of pleural mesothelioma. Lung Cancer. (2025) 210:108852. doi: 10.1016/j.lungcan.2025.108852. PMID: 41308209

[50] Nakajima EC Vellanki PJ Larkins E Chatterjee S Mishra-Kalyani PS Bi Y . FDA approval summary: nivolumab in combination with ipilimumab for the treatment of unresectable Malignant pleural mesothelioma. Clin Cancer Res Off J Am Assoc Cancer Res. (2022) 28:446–51. doi: 10.1158/1078-0432.CCR-21-1466. PMID: 34462287 PMC8810571

