# Supplementary material for: Trop-2 expression is associated with poor survival in pleural mesothelioma
Source: Front Oncol. 2026 May 29;16:1841585. doi: 10.3389/fonc.2026.1841585 (PMC13260006; doi:10.3389/fonc.2026.1841585)
Supplement: Supplementary file 1 [file DataSheet1.docx]

***Supplementary Material***

**Supplementary Table 1**. IHC parameters of Trop-2

**Supplementary Table 2.** Progression-free survival according to clinical characteristics in patients undergoing chemotherapy only.

**Supplementary Table 3**. Overall survival according to clinical characteristics in patients undergoing chemotherapy only.

**Supplementary Table 4.** Clinical characteristics according to sex.

**Supplementary Figure 1.** Selection of patients.

**Supplementary Figure 2**. Progression-free survival (A) and overall survival (B) according to Trop-2 expression in patients undergoing chemotherapy only.

**Supplementary Figure 3.** Therapeutic response evaluation in the whole cohort.

**Supplementary Figure 4**. Therapeutic response evaluation in patients treated with chemotherapy.

| **IHC parameters** | **Total** | **Trop-2 negative** | **Trop-2 positive** |
| --- | --- | --- | --- |
| **Frequency, n (%)** | 60 (100%) | 48 (80.0%) | 12 (20.0%) |
| **Intensity, n (%)** |  |  |  |
| None | 48 (80.0%) | 48 (80.0%) | 0 (0.0%) |
| Positive (+) | 1 (1.7%) | 0 (0.0%) | 1 (1.6%) |
| Positive (++) | 2 (3.3%) | 0 (0.0%) | 2 (3.3%) |
| Positive (+++) | 9 (15%) | 0 (0.0%) | 9 (15%) |
| **Percentage** |  |  |  |
| Median [IQR] | 85.00 [28.0] | 0.0 [0.0] | 85.00 [28.0] |
| 0.0, n (%) | 48 (80.0%) | 48 (80.0%) | 0 (0.0%) |
| 20.0, n (%) | 1 (1.7%) | 0 (0.0%) | 1 (1.7%) |
| 40.0, n (%) | 1 (1.7%) | 0 (0.0%) | 1 (1.7%) |
| 50.0, n (%) | 1 (1.7%) | 0 (0.0%) | 1 (1.7%) |
| 80.0, n (%) | 3 (5.0%) | 0 (0.0%) | 3 (5.0%) |
| 90.0, n (%) | 1 (1.7%) | 0 (0.0%) | 1 (1.7%) |
| 100.0, n (%) | 5 (8.3%) | 0 (0.0%) | 5 (8.3%) |
| **H-score** |  |  |  |
| Median, [IQR] | 220.0 [197.5] | 0.0 [0.0] | 220.0 [197.5] |
| 0, n (%) | 48 (80.0%) | 48 (80.0%) | 0 (0.0%) |
| 60, n (%) | 1 (1.7%) | 0 (0.0%) | 1 (1.7%) |
| 80, n (%) | 3 (5.0%) | 0 (0.0%) | 3 (5.0%) |
| 180, n (%) | 1 (1.7%) | 0 (0.0%) | 1 (1.7%) |
| 200, n (%) | 1 (1.7%) | 0 (0.0%) | 1 (1.7%) |
| 240, n (%) | 2 (3.3%) | 0 (0.0%) | 2 (3.3%) |
| 270, n (%) | 1 (1.7%) | 0 (0.0%) | 1 (1.7%) |
| 300, n (%) | 3 (5.0%) | 0 (0.0%) | 3 (5.0%) |

**Supplementary Table 1. IHC parameters of Trop-2.** Trop-2, Trophoblast cell surface antigen-2. IHC, immunohistochemistry. IQR, interquartile range. The histochemical score (H-score) was calculated by multiplying staining intensity by the percentage of positive cells, yielding a final score ranging from 0 to 300. Statistical significance was defined as p < 0.05, with significant p-values indicated in bold.

|  |  |  |  |  |  | **Progression-free survival, months** | | | **Univariate analysis** | | | **Multivariate analysis** | | |
| --- | --- | --- | --- | --- | --- | --- | --- | --- | --- | --- | --- | --- | --- | --- |
| **Characteristics** | **Total,n** | **Missing,n** | **At risk,n** | **Events,n** | **Censored,n** | **Median** | **95% CI** | **Log-rank** | **HR** | **95% CI** | **p-value** | **HR** | **95% CI** | **p-value** |
| **All** | 43 | 0 | 43 | 38 | 5 | 7.49 | 5.98 — 12.8 |  |  |  |  |  |  |  |
| **Sex, n** |  |  |  |  |  |  |  |  |  |  |  |  |  |  |
| Male | 33 | 0 | 33 | 29 | 4 | 10.61 | 5.98 — 13.31 |  | ref |  |  |  |  |  |
| Female | 10 | 0 | 10 | 9 | 1 | 7.26 | 3.81 — NR | 0.983 | 1.01 | 0.46 — 2.22 | 0.987 |  |  |  |
| **Smoking status, n** |  |  |  |  |  |  |  |  |  |  |  |  |  |  |
| Non-smoker | 21 | 0 | 21 | 19 | 2 | 7.15 | 5.49 — 19.45 |  | ref |  |  |  |  |  |
| Current/former | 22 | 0 | 22 | 19 | 3 | 10.61 | 6.34 — 14 | 0.718 | 0.89 | 0.46 — 1.7 | 0.716 |  |  |  |
| **Passive smoking, n** |  |  |  |  |  |  |  |  |  |  |  |  |  |  |
| Absent | 35 | 0 | 35 | 31 | 4 | 7.26 | 5.95 — 11.53 |  | ref |  |  |  |  |  |
| Present | 8 | 0 | 8 | 7 | 1 | 13.31 | 5.72 — NR | 0.109 | 0.49 | 0.2 — 1.2 | 0.118 |  |  |  |
| **Wood smoke exposure, n** |  |  |  |  |  |  |  |  |  |  |  |  |  |  |
| Absent | 28 | 0 | 28 | 23 | 5 | 10.78 | 7.03 — 14 |  | ref |  |  |  |  |  |
| Present | 15 | 0 | 15 | 15 | 0 | 5.98 | 5.32 — 13.31 | 0.193 | 1.55 | 0.8 — 3.02 | 0.197 |  |  |  |
| **Asbestos exposure, n** |  |  |  |  |  |  |  |  |  |  |  |  |  |  |
| Absent | 30 | 0 | 30 | 27 | 3 | 9.07 | 5.98 — 12.91 |  | ref |  |  |  |  |  |
| Present | 13 | 0 | 13 | 11 | 2 | 7.26 | 5.06 — NR | 0.569 | 1.23 | 0.6 — 2.53 | 0.578 |  |  |  |
| **ECOG, n** |  |  |  |  |  |  |  |  |  |  |  |  |  |  |
| 0-1 | 39 | 0 | 39 | 36 | 3 | 7.49 | 5.98 — 12.81 |  | ref |  |  |  |  |  |
| ≥2 | 4 | 0 | 4 | 2 | 2 | 12.91 | 3.71 — NR | 0.927 | 0.93 | 0.22 — 3.94 | 0.926 |  |  |  |
| **Clinical stage, n** |  |  |  |  |  |  |  |  |  |  |  |  |  |  |
| I-II | 12 | 0 | 12 | 11 | 1 | 12.81 | 10.61 — NR |  | ref |  |  |  |  |  |
| III-IV | 31 | 0 | 31 | 27 | 4 | 7.03 | 5.72 — 11.53 | 0.374 | 1.38 | 0.67 — 2.83 | 0.377 |  |  |  |
| **Histology, n** |  |  |  |  |  |  |  |  |  |  |  |  |  |  |
| Epithelioid | 37 | 0 | 37 | 32 | 5 | 10.61 | 7.2 — 13.31 |  | ref |  |  |  |  |  |
| Biphasic | 1 | 0 | 1 | 1 | 0 | 30.42 | NR — NR |  | 0.34 | 0.04 — 2.53 | 0.291 |  |  |  |
| Non-classified | 1 | 0 | 1 | 1 | 0 | 3.81 | NR — NR |  | 5.07 | 0.62 — 41.21 | 0.129 |  |  |  |
| Sarcomatoid | 4 | 0 | 4 | 4 | 0 | 5.19 | 3.78 — NR | **0.017** | 3.78 | 1.21 — 11.84 | **0.023** |  |  |  |
| **Sarcomatoid histology, n** |  |  |  |  |  |  |  |  |  |  |  |  |  |  |
| No sarcomatoid | 39 | 0 | 39 | 34 | 5 | 10.61 | 6.34 — 13.31 |  | ref |  |  |  |  |  |
| Sarcomatoid | 4 | 0 | 4 | 4 | 0 | 5.19 | 3.78 — NR | **0.015** | 3.78 | 1.21 — 11.84 | **0.023** | 4.54 | 1.24 — 16.56 | **0.021** |
| **Contralateral pleural metastases, n** |  |  |  |  |  |  |  |  |  |  |  |  |  |  |
| Absent | 29 | 0 | 29 | 26 | 3 | 9.07 | 7.03 — 13.31 |  | ref |  |  |  |  |  |
| Present | 14 | 0 | 14 | 12 | 2 | 5.98 | 5.06 — NR | 0.976 | 1.01 | 0.5 — 2.02 | 0.979 |  |  |  |
| **Lung metastases, n** |  |  |  |  |  |  |  |  |  |  |  |  |  |  |
| Absent | 32 | 0 | 32 | 28 | 4 | 9.07 | 5.98 — 12.91 |  | ref |  |  |  |  |  |
| Present | 11 | 0 | 11 | 10 | 1 | 7.49 | 5.72 — NR | 0.553 | 0.8 | 0.37 — 1.7 | 0.562 |  |  |  |
| **NRLN metastases, n** |  |  |  |  |  |  |  |  |  |  |  |  |  |  |
| Absent | 34 | 0 | 34 | 31 | 3 | 8.13 | 5.95 — 12.81 |  | ref |  |  |  |  |  |
| Present | 9 | 0 | 9 | 7 | 2 | 7.49 | 7.26 — NR | 0.277 | 0.62 | 0.26 — 1.5 | 0.288 |  |  |  |
| **Brain metastases, n** |  |  |  |  |  |  |  |  |  |  |  |  |  |  |
| Absent | 42 | 0 | 42 | 37 | 5 | 9.07 | 5.98 — 12.91 |  | ref |  |  |  |  |  |
| Present | 1 | 0 | 1 | 1 | 0 | 7.2 | NR — NR | 0.588 | 1.73 | 0.23 — 13.02 | 0.595 |  |  |  |
| **Adrenal metastases, n** |  |  |  |  |  |  |  |  |  |  |  |  |  |  |
| Absent | 42 | 0 | 42 | 37 | 5 | 9.07 | 6.34 — 12.91 |  | ref |  |  |  |  |  |
| Present | 1 | 0 | 1 | 1 | 0 | 2.4 | NR — NR | NR | 38.99 | 2.44 — 623.45 | 0.01 |  |  |  |
| **Bone metastases, n** |  |  |  |  |  |  |  |  |  |  |  |  |  |  |
| Absent | 35 | 0 | 35 | 31 | 4 | 10.78 | 6.34 — 13.31 |  | ref |  |  |  |  |  |
| Present | 8 | 0 | 8 | 7 | 1 | 5.06 | 3.71 — NR | 0.356 | 1.49 | 0.64 — 3.47 | 0.36 |  |  |  |
| **Liver metastases, n** |  |  |  |  |  |  |  |  |  |  |  |  |  |  |
| Absent | 42 | 0 | 42 | 37 | 5 | 9.07 | 5.98 — 12.91 |  | ref |  |  |  |  |  |
| Present | 1 | 0 | 1 | 1 | 0 | 7.03 | NR — NR | 0.531 | 1.88 | 0.25 — 14.21 | 0.54 |  |  |  |
| **Trop-2 IHC expression** |  |  |  |  |  |  |  |  |  |  |  |  |  |  |
| Negative | 36 | 0 | 36 | 32 | 4 | 10.78 | 7.03 — 13.31 |  | ref |  |  |  |  |  |
| Positive | 7 | 0 | 7 | 6 | 1 | 6.16 | 5.72 — NR | **0.044** | 2.6 | 0.99 — 6.82 | 0.053 | 3.20 | 1.12—9.15 | **0.029** |
| **PLECH score, n** |  |  |  |  |  |  |  |  |  |  |  |  |  |  |
| <3 | 22 | 0 | 22 | 20 | 2 | 10.94 | 5.95 — 19.45 |  | ref |  |  |  |  |  |
| ≥ 3 | 21 | 0 | 21 | 18 | 3 | 7.2 | 5.32 — 11.53 | 0.11 | 1.71 | 0.88 — 3.33 | 0.116 | 1.30 | 0.62 —2.72 | 0.47 |

**Supplementary Table 2. Progression-free survival according to clinical characteristics in patients undergoing chemotherapy only.** mPFS, median progression-free survival. ECOG, Eastern Cooperative Oncology Group. NRLN, non-regional lymph nodes. PLECH, Prognostic Factors in Pleural Mesothelioma Patients Receiving First-Line Chemotherapy including Platelet count (P: +2), high LDH (L: +1), ECOG ≥ 2 (E: +1), Chest pain at diagnosis (C: +2), and non-epithelioid Histology (H: +1). TROP-2, Trophoblast cell surface antigen-2. IHC, immunohistochemistry. aHR, adjusted hazard ratio. NR, not reached. Statistical significance was defined as p < 0.05, with significant p-values indicated in bold.

|  |  |  |  |  |  | **Overall survival, months** | | | **Univariate analysis** | | | **Multivariate analysis** | | |
| --- | --- | --- | --- | --- | --- | --- | --- | --- | --- | --- | --- | --- | --- | --- |
| **Characteristics** | **Total** | **Missing,n** | **At risk,n** | **Events,n** | **Censored,n** | **Median** | **95% CI** | **Log-rank** | **HR** | **95% CI** | **p-value** | **HR** | **95% CI** | **p-value** |
| All | 43 | 0 | 43 | 23 | 20 | 16.4 | 10.12 — NR |  |  |  |  |  |  |  |
| **Sex, n** |  |  |  |  |  |  |  |  |  |  |  |  |  |  |
| Male | 33 | 0 | 33 | 17 | 16 | 31.9 | 12.09 — NR |  | ref |  |  |  |  |  |
| Female | 10 | 0 | 10 | 6 | 4 | 8.77 | 5.06 — NR | 0.241 | 1.75 | 0.68 — 4.49 | 0.245 |  |  |  |
| **Smoking status, n** |  |  |  |  |  |  |  |  |  |  |  |  |  |  |
| Non-smoker | 21 | 0 | 21 | 15 | 6 | 10.12 | 7.98 — NR |  | ref |  |  |  |  |  |
| Current/former | 22 | 0 | 22 | 8 | 14 | 116.93 | 16 — NR | **0.019** | 0.35 | 0.14 — 0.87 | **0.024** |  |  |  |
| **Passive smoking, n** |  |  |  |  |  |  |  |  |  |  |  |  |  |  |
| Absent | 35 | 0 | 35 | 20 | 15 | 16 | 9.56 — NR |  | ref |  |  |  |  |  |
| Present | 8 | 0 | 8 | 3 | 5 | 116.93 | 16.36 — NR | 0.075 | 0.29 | 0.07 — 1.24 | 0.094 |  |  |  |
| **Wood smoke exposure, n** |  |  |  |  |  |  |  |  |  |  |  |  |  |  |
| Absent | 28 | 0 | 28 | 17 | 11 | 16 | 9.56 — NR |  | ref |  |  |  |  |  |
| Present | 15 | 0 | 15 | 6 | 9 | 38.93 | 9.43 — NR | 0.332 | 0.63 | 0.25 — 1.62 | 0.337 |  |  |  |
| **Asbestos exposure, n** |  |  |  |  |  |  |  |  |  |  |  |  |  |  |
| Absent | 30 | 0 | 30 | 16 | 14 | 16.36 | 12.09 — NR |  | ref |  |  |  |  |  |
| Present | 13 | 0 | 13 | 7 | 6 | 31.9 | 7.26 — NR | 0.551 | 1.32 | 0.53 — 3.25 | 0.55 |  |  |  |
| **ECOG, n** |  |  |  |  |  |  |  |  |  |  |  |  |  |  |
| 0-1 | 39 | 0 | 39 | 23 | 16 | 16.0 | 9.56 — 43.4 |  | ref |  |  |  |  |  |
| ≥2 | 4 | 0 | 4 | 0 | 4 | NR | NR — NR | 0.167 | NR | NR — NR | 0.998 |  |  |  |
| **Clinical stage, n** |  |  |  |  |  |  |  |  |  |  |  |  |  |  |
| I-II | 12 | 0 | 12 | 6 | 6 | 33.71 | 13.57 — NR |  | ref |  |  |  |  |  |
| III-IV | 31 | 0 | 31 | 17 | 14 | 16.36 | 8.77 — NR | 0.544 | 1.34 | 0.52 — 3.47 | 0.543 |  |  |  |
| **Histology, n** |  |  |  |  |  |  |  |  |  |  |  |  |  |  |
| Epithelioid | 37 | 0 | 37 | 19 | 18 | 24.77 | 12.09 — NR |  | ref |  |  |  |  |  |
| Biphasic | 1 | 0 | 1 | 1 | 0 | 38.93 | NR — NR |  | 0.86 | 0.11 — 6.55 | 0.885 |  |  |  |
| Non-classified | 1 | 0 | 1 | 0 | 1 | NR | NR — NR |  | NR | NR — NR | 0.999 |  |  |  |
| Sarcomatoid | 4 | 0 | 4 | 3 | 1 | 7.26 | 4.11 — NR | 0.12 | 4.43 | 1.2 — 16.39 | **0.026** |  |  |  |
| **Sarcomatoid histology, n** |  |  |  |  |  |  |  |  |  |  |  |  |  |  |
| No sarcomatoid | 39 | 0 | 39 | 20 | 19 | 24.77 | 13.57 — NR |  | ref |  |  |  |  |  |
| Sarcomatoid | 4 | 0 | 4 | 3 | 1 | 7.27 | 4.11 — NR | **0.016** | 4.43 | 1.2 — 16.39 | **0.026** | 4.86 | 1.11 — 21.16 | **0.034** |
| **Contralateral pleural metastases, n** |  |  |  |  |  |  |  |  |  |  |  |  |  |  |
| Absent | 29 | 0 | 29 | 14 | 15 | 16 | 10.12 — NR |  | ref |  |  |  |  |  |
| Present | 14 | 0 | 14 | 9 | 5 | 24.77 | 7.26 — NR | 0.986 | 1.01 | 0.41 — 2.47 | 0.983 |  |  |  |
| **Lung metastases, n** |  |  |  |  |  |  |  |  |  |  |  |  |  |  |
| Absent | 32 | 0 | 32 | 17 | 15 | 16.36 | 12.09 — NR |  | ref |  |  |  |  |  |
| Present | 11 | 0 | 11 | 6 | 5 | 116.93 | 7.26 — NR | 0.428 | 0.66 | 0.24 — 1.86 | 0.437 |  |  |  |
| **NRLN metastases, n** |  |  |  |  |  |  |  |  |  |  |  |  |  |  |
| Absent | 34 | 0 | 34 | 19 | 15 | 16.36 | 10.12 — NR |  | ref |  |  |  |  |  |
| Present | 9 | 0 | 9 | 4 | 5 | 31.9 | 7.26 — NR | 0.89 | 0.93 | 0.31 — 2.75 | 0.895 |  |  |  |
| **Brain metastases, n** |  |  |  |  |  |  |  |  |  |  |  |  |  |  |
| Absent | 42 | 0 | 42 | 23 | 19 | 16.36 | 10.12 — NR |  | ref |  |  |  |  |  |
| Present | 1 | 0 | 1 | 0 | 1 | NR | NR — NR | 0.547 | NR | 0 — NR | 0.998 |  |  |  |
| **Adrenal metastases, n** |  |  |  |  |  |  |  |  |  |  |  |  |  |  |
| Absent | 42 | 0 | 42 | 23 | 19 | 16.36 | 10.12 — NR |  | ref |  |  |  |  |  |
| Present | 1 | 0 | 1 | 0 | 1 | NR | NR — NR | 0.625 | NR | 0 — NR | 0.998 |  |  |  |
| **Bone metastases, n** |  |  |  |  |  |  |  |  |  |  |  |  |  |  |
| Absent | 35 | 0 | 35 | 17 | 18 | 31.9 | 13.57 — NR |  | ref |  |  |  |  |  |
| Present | 8 | 0 | 8 | 6 | 2 | 9.56 | 4.11 — NR | 0.069 | 2.37 | 0.91 — 6.13 | 0.076 |  |  |  |
| **Liver metastases, n** |  |  |  |  |  |  |  |  |  |  |  |  |  |  |
| Absent | 42 | 0 | 42 | 22 | 20 | 24.77 | 12.09 — NR |  | ref |  |  |  |  |  |
| Present | 1 | 0 | 1 | 1 | 0 | 10.12 | NR — NR | 0.398 | 2.34 | 0.3 — 18.09 | 0.414 |  |  |  |
| **Trop-2 IHC expression** |  |  |  |  |  |  |  |  |  |  |  |  |  |  |
| Negative | 36 | 0 | 36 | 18 | 18 | 31.9 | 13.57 — NR |  | ref |  |  |  |  |  |
| Positive | 7 | 0 | 7 | 5 | 2 | 8.38 | 7.26 — NR | **0.011** | 3.88 | 1.26 — 11.92 | **0.018** | 6.07 | 1.73 — 21.24 | **0.004** |
| **PLECH score, n** |  |  |  |  |  |  |  |  |  |  |  |  |  |  |
| <3 | 22 | 0 | 22 | 10 | 12 | 43.4 | 16 — NR |  | ref |  |  |  |  |  |
| ≥ 3 | 21 | 0 | 21 | 13 | 8 | 10.12 | 7.26 — NR | **0.021** | 2.77 | 1.13 — 6.8 | **0.026** | 2.51 | 0.97 — 6.51 | 0.056 |

**Supplementary Table 3. Overall survival according to clinical characteristics in patients undergoing chemotherapy only.** mOS, median overall survival. ECOG PS, Eastern Cooperative Oncology Group Performance Status. NRLN, non-regional lymph nodes. PLECH, Prognostic Factors in Pleural Mesothelioma Patients Receiving First-Line Chemotherapy including Platelet count (P: +2), high LDH (L: +1), ECOG ≥ 2 (E: +1), Chest pain at diagnosis (C: +2), and non-epithelioid Histology (H: +1). Trop-2, Trophoblast cell surface antigen-2. IHC, immunohistochemistry. aHR, adjusted hazard ratio. NR, not reached. Statistical significance was defined as p < 0.05, with significant p-values indicated in bold.

| **Clinical characteristics** | **Overall** | **Male** | **Female** | ***p* value** |
| --- | --- | --- | --- | --- |
|  | **n (%)**  60 (100) | **n (%)**  46 (76.7) | **n (%)**  14 (23.3) |  |
| **Age, (median [IQR])** | 63.50 [18.25] | 63.50 [17.25] | 63.00 [21.00] | 0.668 |
| **Trop-2 IHC expression (%)** |  |  |  |  |
| Negative | 48 (80.0) | 39 (84.8) | 9 (64.3) |  |
| Positive | 12 (20.0) | 7 (15.2) | 5 (35.7) | 0.195 |
| **Smoking status, n (%)** |  |  |  |  |
| Current/former | 32 (53.3) | 29 (63.0) | 3 (21.4) |  |
| Non-smoker | 28 (46.7) | 17 (37.0) | 11 (78.6) | **0.013*** |
| **Passive smoking, n (%)** |  |  |  |  |
| Absent | 50 (83.3) | 41 (89.1) | 9 (64.3) |  |
| Present | 10 (16.7) | 5 (10.9) | 5 (35.7) | 0.076 |
| **Wood smoke exposure, n (%)** |  |  |  |  |
| Absent | 43 (71.7) | 33 (71.7) | 10 (71.4) |  |
| Present | 17 (28.3) | 13 (28.3) | 4 (28.6) | 1.000* |
| **Asbestos exposure, n (%)** |  |  |  |  |
| Absent | 39 (65.0) | 30 (65.2) | 9 (64.3) | 1.000 |
| Present | 21 (35.0) | 16 (34.8) | 5 (35.7) |  |
| **ECOG PS, n (%)** |  |  |  |  |
| 0-1 | 54 (90.0) | 41 (89.1) | 13 (92.9) |  |
| ≥2 | 6 (10.0) | 5 (10.9) | 1 (7.1) | 1.000* |
| **Clinical stage, n (%)** |  |  |  |  |
| I-II | 16 (26.7) | 14 (30.4) | 2 (14.3) |  |
| III-IV | 44 (73.3) | 32 (69.6) | 12 (85.7) | 0.314* |
| **Histology, n (%)** |  |  |  |  |
| Epithelioid | 51 (85.0) | 40 (87.0) | 11 (78.6) |  |
| Biphasic | 1 (1.7) | 1 (2.2) | 0 (0.0) |  |
| Sarcomatoid | 5 (8.3) | 4 (8.7) | 1 (7.1) |  |
| Non-classified | 3 (5.0) | 1 (2.2) | 2 (14.3) | 0.311 |
| **Pleural effusion, n (%)** |  |  |  |  |
| Absent | 36 (60.0) | 30 (65.2) | 6 (42.9) |  |
| Present | 24 (40.0) | 16 (34.8) | 8 (57.1) | 0.212 |
| **Metastases sites, n (%) ***** |  |  |  |  |
| Contralateral pleural | 18 (30.0) | 14 (30.4) | 4 (28.6) | 1.000* |
| Lung | 16 (26.7) | 13 (28.3) | 3 (21.4) | 0.872* |
| Brain | 1 (1.7) | 1 (2.2) | 0 (0.0) | 1.000* |
| Adrenal | 2 (3.3) | 2 (4.3) | 0 (0.0) | 1.000* |
| Bone | 11 (18.3) | 10 (21.7) | 1 (7.1) | 0.430* |
| Liver | 2 (3.3) | 1 (2.2) | 1 (7.1) | 0.415* |
| **First-line treatment, n (%)** |  |  |  |  |
| Chemotherapy only | 43 (71.7) | 33 (71.7) | 10 (71.4) |  |
| Multimodal therapy** | 7 (11.7) | 6 (13.0) | 1 (7.1) |  |
| Surgery only | 2 (3.3) | 2 (4.3) | 0 (0.0) |  |
| Best supportive care | 8 (13.3) | 5 (10.9) | 3 (21.4) | 0.609 |
| **PLECH score, (median [IQR])** | 3.00 [3.00] | 3.00 [2.00] | 2.50 [3.75] | 0.922 |
| **PLECH score, n (%)** |  |  |  |  |
| <3 | 29 (48.3) | 22 (47.8) | 7 (50.0) |  |
| ≥ 3 | 31 (51.7) | 24 (52.2) | 7 (50.0) | 1.000 |

**Supplementary Table 4. Clinical characteristics according to sex.** ECOG, Eastern Cooperative Oncology Group. NRLN, non-regional lymph nodes. PLECH, Prognostic Factors in Pleural Mesothelioma Patients Receiving First-Line Chemotherapy including Platelet count (P: +2), high LDH (L: +1), ECOG ≥ 2 (E: +1), Chest pain at diagnosis (C: +2), and non-epithelioid Histology (H: +1). Trop-2, Trophoblast cell surface antigen-2. IHC, immunohistochemistry. IQR, interquartile range. **Multimodal therapy included chemotherapy, radiotherapy and surgery. *** Metastases sites may not sum 60 as each patient can have more than 1 metastatic site. Statistical significance was defined as p < 0.05, with significant p-values indicated in bold.


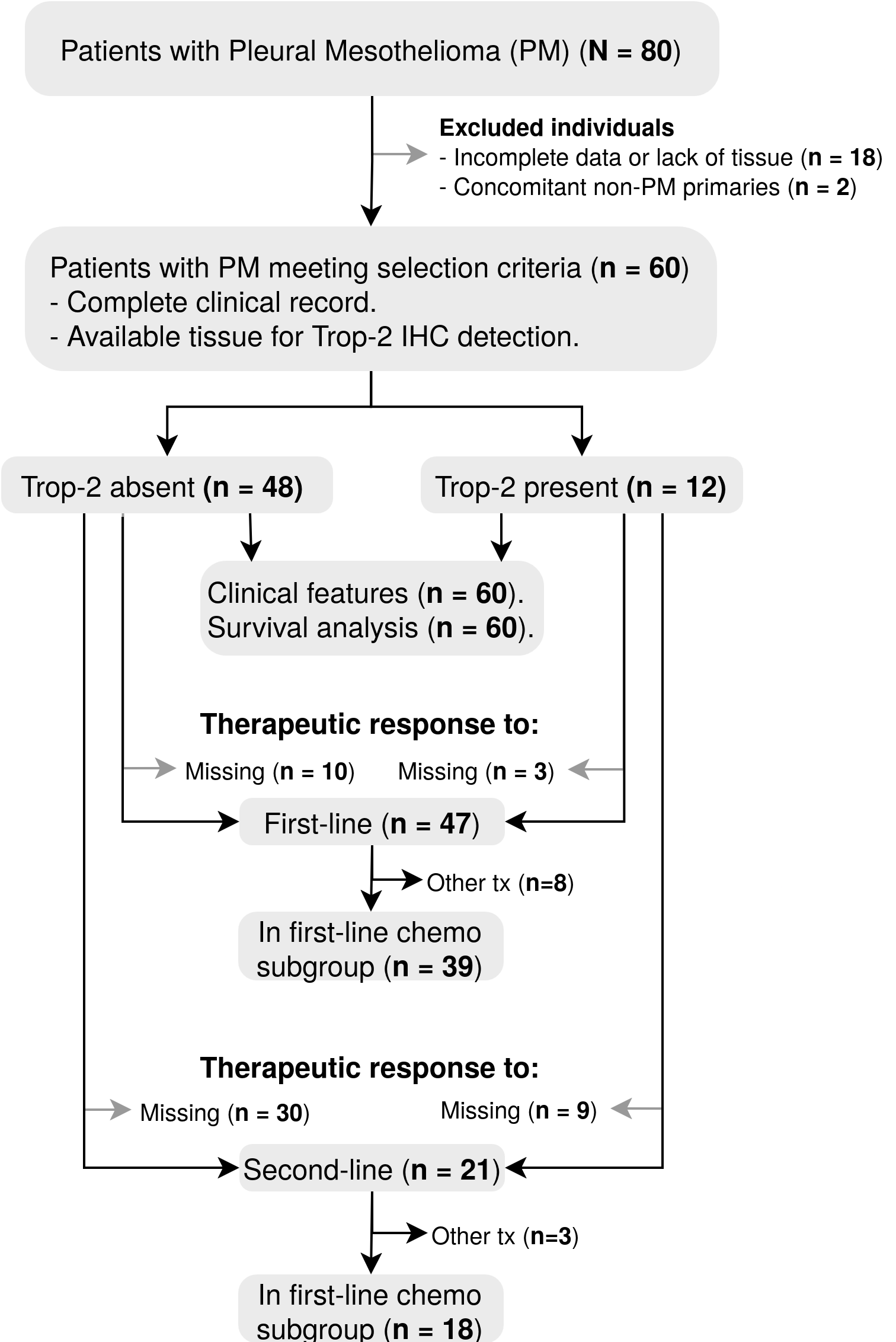


**Supplementary Figure 1. Selection of patients.** Trop-2, Trophoblast surface antigen 2. PM, Pleural Mesothelioma. tx, treatment. chemo, chemotherapy.


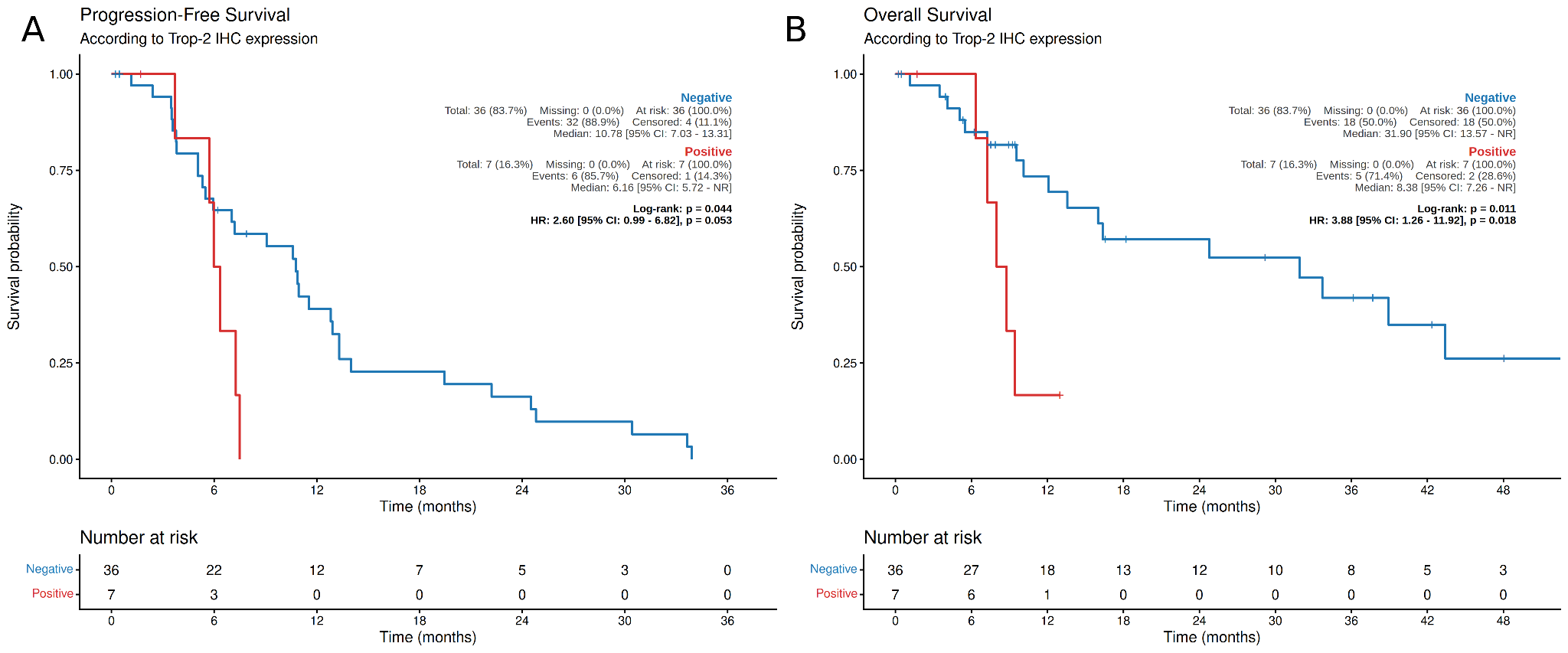


**Supplementary Figure 2.** **Progression-free survival (A) and overall survival (B) according to Trop-2 expression in patients undergoing chemotherapy only.** HR: hazard ratio; CI: confidence interval; PFS: progression-free survival; OS: overall survival. TROP-2, Trophoblast cell surface antigen-2. IHC, immunohistochemistry. HR, hazard ratio. CI, confidence interval.


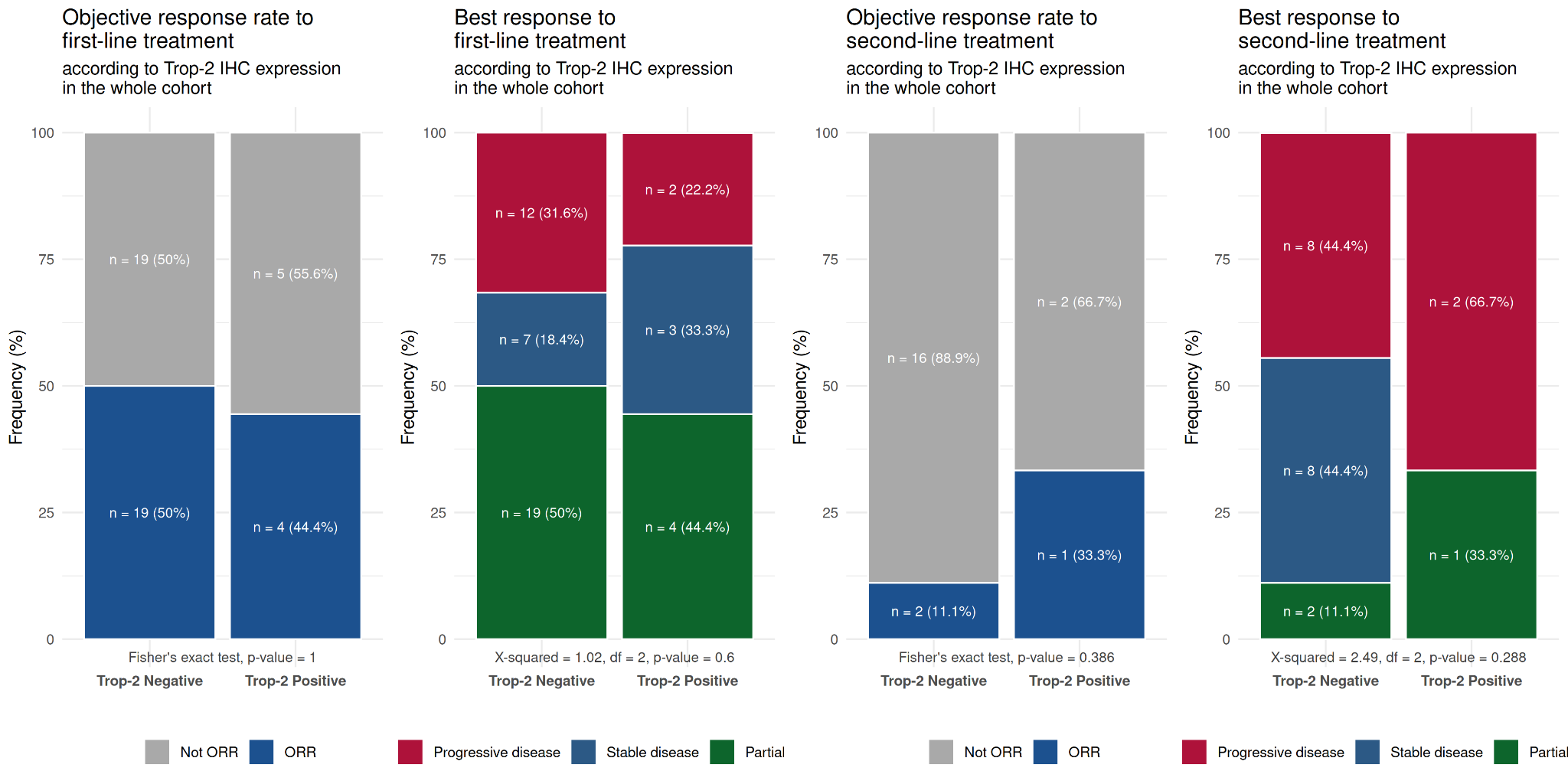


**Supplementary Figure 3. Therapeutic response evaluation in the whole cohort.** ORR, objective response rate. Statistical differences were determined using chi-squared test or Fisher’s exact test, and statistical significance was set at a p-value <0.05.


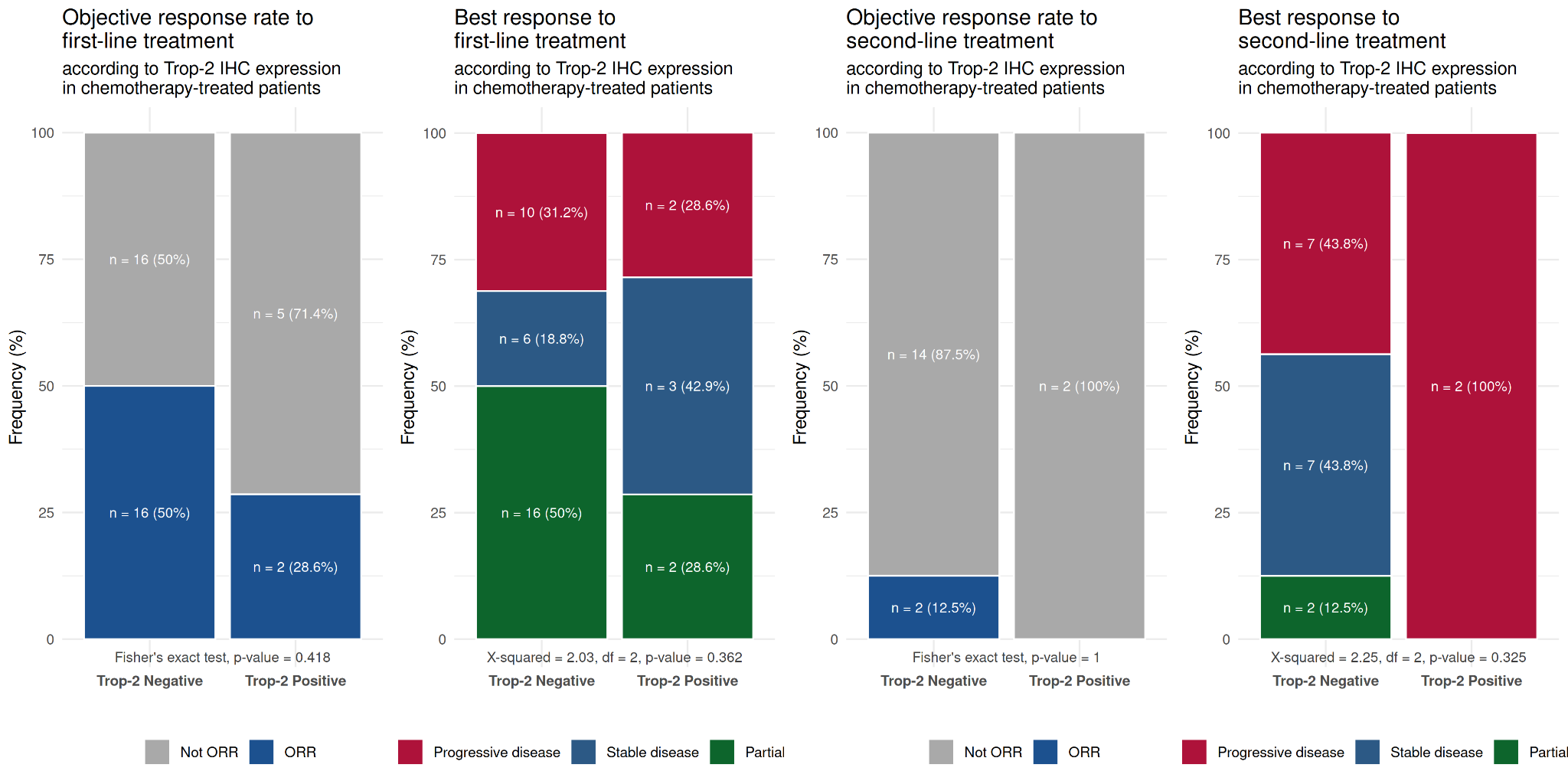


**Supplementary Figure 4. Therapeutic response evaluation in the whole cohort.** ORR, objective response rate. Statistical differences were determined using chi-squared test or Fisher’s exact test, and statistical significance was set at a p-value <0.05.
